# A multiobjective human evolutionary optimization algorithm for complex engineering problems

**DOI:** 10.1038/s41598-025-34467-5

**Published:** 2026-01-08

**Authors:** D. Tarunika, Ashish Sharma

**Affiliations:** Department of Electronics and Communication Engineering, Manipal Institute of Technology Bengaluru, Manipal Academy of Higher Education, Manipal, India

**Keywords:** Dynamic archive, Human evolutionary dynamics, Multi-objective optimization, Pareto optimality, Roulette–Wheel Selection, Engineering, Mathematics and computing

## Abstract

Multi-objective optimization problems (MOPs) demand algorithms that effectively balance convergence, diversity, and computational efficiency. To address this challenge, a novel Multi-Objective Human Evolutionary Optimization Algorithm (MOHEOA) is proposed, inspired by the dynamics of human societal evolution. MOHEOA structures the search process into two adaptive phases: human exploration and human development, integrating a fixed-size dynamic archive to maintain and utilize non-dominated Pareto solutions. The algorithm begins with a logistic chaos mapping for population initialization, ensuring robust diversity. During the development phase, individuals are classified into leaders, explorers, followers, and losers, each employing specialized strategies tailored for multi-objective search. A roulette-wheel selection mechanism dynamically selects leaders from the archive, optimizing the trade-off between exploration and exploitation. To validate MOHEOA’s performance, extensive experiments on twenty-three benchmark test functions and four real-world engineering design problems are conducted. Comparative evaluations against state-of-the-art multi-objective algorithms demonstrate that MOHEOA consistently outperforms competitors in convergence speed, solution diversity, and Pareto optimality. The algorithm’s robustness and adaptability make it a compelling choice for complex optimization tasks. For reproducibility and further research, the MATLAB implementation of MOHEOA is publicly available at: https://github.com/swatzash/MOHEOA.

## Introduction

Over the years, metaheuristic approaches have gained significant attention as effective tools for addressing real-world challenges. They are derived from evolutionary algorithms^1,2^, swarm intelligence (SI)^3,4^, physics-based approaches^5,6^, and human-inspired methods^7,8^, etc. Their appeal lies in their computational efficiency and simplicity, making them widely applicable in various engineering problems^9–11^. Engineering problems are often nonlinear and lack simple analytical solutions. A particularly challenging aspect of solving these problems lies in multi-objective optimization, which requires balancing and optimizing multiple, often competing, objectives simultaneously. Addressing these complexities demands the use of sophisticated optimization techniques capable of identifying effective and efficient solutions^12^. In single-objective optimization, it is relatively simple to identify the superior solution, usually resulting in a single optimal outcome. However, in multi-objective optimization, comparing solutions is more complex due to the need to balance multiple conflicting objectives. As a result, the process yields a set of solutions that address these objectives simultaneously rather than a single definitive solution^13,14^. The primary difficulty in multi-objective optimization arises from the need to balance conflicting goals effectively. There are three predominant strategies to tackle multi-objective problems: priori, posteriori, and interactive approaches^15,16^. In the priori method, the problem is simplified to a single-objective one by assigning weights that represent the relative importance of each objective^17^. On the other hand, the posteriori method involves exploring possible solutions and selecting the most appropriate one based on specific requirements^18^. Unlike single-objective optimization, where a single optimal solution exists, multi-objective optimization yields a set of trade-off solutions. These solutions form what is known as the *Pareto front*, which represents compromises between objectives. Generating a *Pareto front* is a time-intensive process, as it requires the identification of multiple points to approximate the front accurately. Furthermore, even when precise solutions are obtained, they may not distribute evenly across the Pareto front. High-dimensional problems introduce additional complexity, as their Pareto fronts often form intricate hypersurfaces, making them particularly challenging to solve^19^. Stochastic methods for multi-objective optimization are advantageous because they avoid local optima and do not rely on gradient information, making them suitable for solving practical problems^20^. Multi-objective optimization finds applications across various fields, tackling complex challenges in diverse industries. In addition to its well-known uses in renewable energy^21^, civil^22^, mechanical^23^, and it is also extensively applied in bioinformatics for genomic data analysis^24,25^, software engineering for resource allocation^26^, and industrial engineering^27^. Beyond these, the technique proves valuable in areas such as telecommunications^28^, automotive design^29^, robotics^30^, routing and scheduling^31^, and healthcare^32^. Its ability to simultaneously optimize multiple conflicting objectives makes it an essential tool for addressing real-world problems that require balancing trade-offs in various disciplines. Yang suggested that algorithms cannot be categorized as inherently good or bad; rather, their suitability depends on the specific optimization problem at hand^33^. Achieving an optimal balance between exploration, exploitation, convergence, solution diversity, and computational efficiency remains a significant challenge for any single algorithm when addressing multi-objective optimization problems. This underscores the ongoing need for the development of innovative meta-heuristics capable of effectively handling the complexities of such problems. Recently, Lian et al. developed a new meta-heuristic algorithm inspired by human evolution, the Human Evolutionary Optimization Algorithm (HEOA)^8^. HEOA consists of two phases: human exploration and human development. In the development phase, the population is divided into leaders, explorers, followers, and losers, each employing distinct strategies. Lian et al. provides a comprehensive overview of HEOA’s creation, validation, and application in complex optimization problems^8^. One of the reasons for the success of the HEOA is its ability to integrate multiple strategies into a single optimizer. HEOA combines stochastic search based on population dynamics and trajectory with adaptive, jumping, and Levy distributional strategies. These strategies allow HEOA to explore the search space effectively, from local regions to the entire space. Points selected from these strategies are evaluated in the objective function, aiding the algorithm in finding globally optimal solutions. This approach demonstrates strong theoretical potential and can be especially beneficial for multi-objective optimization problems. This paper presents the first comprehensive overview of the evolution of the HEOA into the Multi-Objective Human Evolutionary Optimization Algorithm (MOHEOA).

The motivation for selecting the Human Evolutionary Optimization Algorithm (HEOA) as the basis for this study stems from its unique dual-phase search strategy inspired by human societal evolution, which effectively balances exploration and exploitation. Unlike many single-objective metaheuristics, HEOA employs adaptive role-based population dynamics and logistic chaos mapping to enhance diversity and convergence simultaneously. Notably, prior to this work, no multi-objective version of HEOA had been developed or evaluated. The original single-objective HEOA demonstrated superior performance across numerous benchmark optimization problems, outperforming several state-of-the-art algorithms. Given this empirical success and the critical importance of balancing convergence and diversity in multi-objective optimization, extending HEOA to a multi-objective framework was both a logical and promising direction. This approach aims to leverage its proven strengths in maintaining solution quality while addressing the challenges inherent in multi-objective problems, thereby filling an existing gap in the literature and advancing optimization methodologies. The algorithm’s performance will be evaluated using a range of benchmark test functions, including six from the widely-used ZDT test functions^34^, ten challenging functions from the CEC2009 group^35^, and recently developed yet most challenging eight IMPO test functions^36^. In addition, the optimizer’s effectiveness will be assessed on three constrained real-world engineering problems, providing a thorough evaluation across diverse scenarios. Prominent examples of such optimization techniques include the Non-dominated sorting genetic algorithm II (NSGA-II), Multi-objective particle swarm optimization (MOPSO), and the Multi-objective evolutionary algorithm based on decomposition (MOEA/D).

The existing literature shows that these algorithms are capable of effectively approximating the true Pareto optimal solutions for multi-objective problems. However, the No Free Lunch (NFL) theorem^37^ presents a key argument: no single optimization method can universally solve all optimization problems. This principle states that an optimizer’s superior performance on one set of problems does not guarantee the same success on another set. The NFL theorem has influenced many studies in the field, encouraging researchers to adapt existing techniques for new problem classes or to develop entirely new optimization algorithms. This concept also forms the foundation and inspiration for the current work, where we introduce an innovative multi-objective optimization algorithm. The proposed algorithm aims to address the limitations of existing techniques, with its primary contributions outlined in this study. The key contributions of this research can be summarized as follows:

- The HEOA algorithm incorporates an archive to store and maintain non-dominated solutions.
- A grid-based mechanism is integrated into the HEOA to enhance the quality of solutions in the archive.
- A leader selection mechanism has been introduced to guide the update and replacement of solutions in the archive.
- The multi-objective version of HEOA is presented, incorporating the three operators mentioned above.

Performance comparisons were made against six state-of-the-art optimization methods, including Multi-objective Particle Swarm Optimization (MOPSO)^38^, Non-dominated Sorting Genetic Algorithm-II (NSGA-II)^39^, Strength Pareto Evolutionary Algorithm (SPEA-2)^40^ Multi-objective Evolutionary Algorithm based on Decomposition (MOEA/D)^41^, and recently developed Multi-objective Chimp optimization Algorithm (MOChOA)^42^. The evaluation employed four widely recognized performance metrics, demonstrating that MOHEOA is a competitive and promising approach for tackling multi-objective optimization challenges. The structure of this paper is organized as follows: Section 2 introduces the foundational principles of multi-objective optimization along with a review of related studies. Section 3 outlines the concepts of the original HEOA and details the proposed MOHEOA algorithm. Section 4 provides a detailed analysis of the proposed MOHEOA, outlining the various stages of its development and the underlying mechanisms that drive its optimization performance. Section 5 presents the results and discussion using various performance metrics and statistical parameters to evaluate and compare the effectiveness of the proposed MOHEOA. Section 6 evaluates MOHEOA’s performance on four constrained multi-objective engineering design problems and compares it with other established techniques. Finally, Section 7 provides conclusions and suggests directions for future research.

## Background

This section introduces the principles of multi-objective optimization and explores the current methods utilized in the domain of meta-heuristics.

### Multi-objective optimization

Multi-objective optimization tackles problems requiring simultaneous optimization of several competing objectives. The conventional representation frames this as an optimization problem aimed at maximization, defined by:

1 $$\begin{aligned} \begin{aligned} \text {Maximize: }&F(x) = \big ( f_1(x), f_2(x), \dots , f_o(x) \big ) \\ \text {Subject to: }&g_i(x) \ge 0, \quad \forall i \in \{1, 2, \dots , m\} \\&h_i(x) = 0, \quad \forall i \in \{1, 2, \dots , p\} \\&x_i \in [L_i, U_i], \quad \forall i \in \{1, 2, \dots , n\} \end{aligned} \end{aligned}$$

here, $n$ denotes the variable count, $o$ the number of objectives, $m$ the inequality constraints, and $p$ the equality constraints. The functions $g_i(x)$ and $h_i(x)$ represent the $i$-th inequality and equality constraints, respectively, while $[L_i, U_i]$ defines the feasible bounds for each variable $x_i$.

In single-objective optimization, solutions are easily compared, with $X$ being better than $Y$ if $X > Y$. In multi-objective optimization, solutions cannot be directly compared due to multiple criteria. A solution dominates another if it is better or equal in all objectives and strictly better in at least one. In the context of maximization problems, the concept of Pareto dominance can be formally defined as follows (assuming maximization without loss of generality):

1. In multi-objective optimization, Pareto dominance is a way of comparing two solutions^43^. Let $\textbf{x} = (x_1, x_2, \dots , x_k)$ and $\textbf{y} = (y_1, y_2, \dots , y_k)$ be two vectors. Vector $\textbf{x}$ is said to dominate vector $\textbf{y}$ (denoted as $\textbf{x} \succ \textbf{y}$) if and only if:
  2 $$\begin{aligned} \forall i \in \{1, 2, \dots , k\}, \quad f(x_i) \ge f(y_i) \quad \text {and} ~~ \exists i \in \{1, 2, \dots , k\}, \quad f(x_i) > f(y_i) \end{aligned}$$
2. A solution $\textbf{x} \in X$ is considered Pareto optimal if and only if:
  $$\forall \textbf{y} \in X, \quad F(\textbf{y}) \not \prec F(\textbf{x})$$
  where $F(\textbf{y})$ represents the objective values of solution $\textbf{y}$, and $F(\textbf{x})$ denotes the objective values of solution $\textbf{x}$.
3. The set of all non-dominated solutions to a problem is known as the Pareto optimal set, which can be defined as:
  $$P_s := \{ \textbf{x}, \textbf{y} \in X \mid \exists F(\textbf{y}) \prec F(\textbf{x}) \}$$
4. The Pareto optimal front is the set of objective function values corresponding to the Pareto optimal solutions. It is defined as:
  $$P_f := \{ F(\textbf{x}) \mid \textbf{x} \in P_s \}$$

**Fig. 1:**
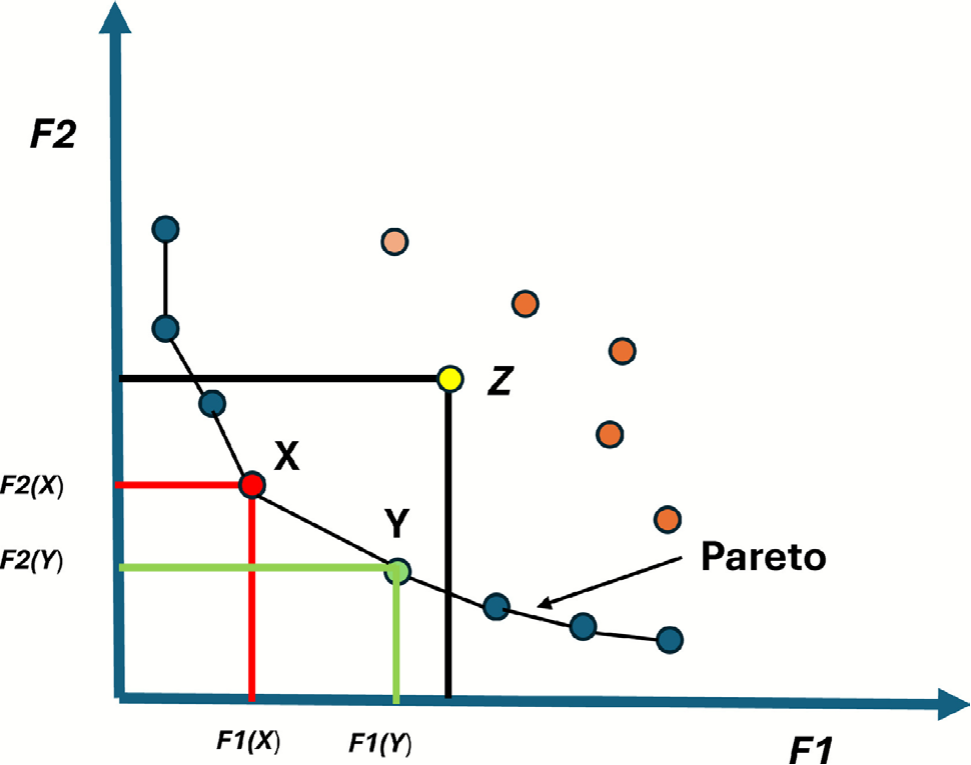
In the two-objective space, solutions X and Y both outperform and dominate solution Z.

Figure 1 illustrates the concept of dominated and non-dominated solutions in a multi-objective optimization (MOP) problem with two objectives. The objective is to minimize both objective functions. The connected curve represents the Pareto front (PF), which includes all Pareto-optimal solutions. As depicted in Fig. 1, solution $Z$ has higher values for both $f_1$ and $f_2$ when compared to solutions $X$ and $Y$. Thus, solutions $X$ and $Y$ dominate solution $Z$. However, since neither $X$ nor $Y$ dominates the other, they are considered non-dominated solutions.

### Multi-objective meta-heuristics

Multi-objective optimization algorithms try to find the best possible solutions (Pareto-optimal) while keeping them well-spread^43^. Earlier approaches aggregated objectives into a single one but had drawbacks: even weight distribution didn’t ensure evenly spread Pareto solutions, and non-convex regions of the Pareto front couldn’t be identified due to restrictions on weights^44^. Several studies in the literature have attempted to enhance this approach. For instance, advancements in multi-objective optimization have enhanced the adaptive weighted sum method to overcome earlier limitations^45,46^. Despite these efforts, the core issues with the aggregation method remain unresolved. Moreover, these techniques require multiple executions to approximate the entire set of Pareto-optimal solutions, as only a single optimal solution is achieved in each iteration. Heuristic methods face significant challenges in optimization due to constraints, uncertainty, multiple objectives, and dynamic changes. Dynamic environments require adaptive operators to track shifting optima and maintain solution viability. Managing uncertainty and distinguishing feasible solutions are essential for convergence to optimal outcomes. Surrogate models are used to reduce computational costs, while Pareto dominance operators help identify optimal solutions in multi-objective problems. Effective heuristics must address all objectives, maintain a well-updated archive of non-dominated solutions, and ensure uniform distribution across the Pareto front for balanced optimization. Multi-objective optimization enables faster convergence by facilitating information sharing among solutions, identifying the complete Pareto front in a single run, and supporting testing across various design settings. It also helps metaheuristics address challenges such as managing infeasible regions, local Pareto fronts, solution diversity, and isolated optimal solutions. While these methods require more advanced techniques and must tackle conflicting objectives, many well-known algorithms have been successfully adapted to perform effectively in multi-objective optimization. The subsequent sections provide a concise overview of the most prominent and recent methods.

One of the most famous and widely used multi-objective metaheuristic methods was introduced by^39^, known as Non-dominated Sorting Genetic Algorithm II (NSGA-II). This method uses non-dominated sorting and a niching operator to achieve optimal results. A random population is generated, grouped by non-dominated sorting, and supported by another population for selection, mutation, and recombination. Each iteration forms a new population ranked by sorting, with selection focused on maintaining diversity and quality. The cycle continues iteratively until accurate results are achieved. The Multi-Objective Particle Swarm Optimization (MOPSO) algorithm is the second most popular multi-objective metaheuristic developed by^38^. MOPSO uses particles to explore the search space for solutions, tracking the best positions they find. However, MOPSO aims to find multiple optimal solutions, not just one. Particles update their positions based on their best solutions and one of the swarm’s best solutions. An external archive stores Pareto-optimal solutions, and a mutation mechanism is used to increase randomness and improve diversity. The Multi-objective Evolutionary Algorithm based on Decomposition (MOEA/D), proposed by^41^, has gained significant popularity for addressing multi-objective optimization problems. MOEA/D addresses multi-objective optimization by dividing it into smaller sub-problems, optimized cooperatively using neighborhood relationships based on Euclidean distance between weight vectors. High-quality solutions for one sub-problem are shared with neighbors, accelerating convergence. Compared to NSGA-II, MOEA/D is computationally more efficient. Multi-objective ant colony optimization is another famous multi-objective meta-heuristic algorithm inspired by ants’ natural ability to find the shortest path to food using pheromone trails^47^. Ants communicate indirectly by leaving pheromones, which guide others in the colony. This approach incorporates pheromone deposition, trail updates, de-centralized decision-making, and external interventions to optimize solutions effectively. The multi-objective grey wolf optimization (MOGWO), introduced by^48^, is an effective and efficient algorithm for solving Multi-Objective Problems (MOPs). It mimics the hunting behaviour of grey wolves, using their movement patterns to explore search spaces. Its strength lies in its dynamic, parameter-controlled operations, which balance exploration and exploitation, preventing local optima stagnation. MOGWO excels at exploring search spaces and finding optimal solutions through the use of three key search agents, alpha, beta, and delta wolves. Over the years, several advanced algorithms have been developed to effectively address multi-objective optimization problems, each offering unique features and superior performance. Among them is the Strength Pareto Evolutionary Algorithm (SPEA)^40^, which utilizes Pareto dominance to allocate fitness to individuals and reduces the number of non-dominated solutions in external archives through clustering. Additionally, the Multi-objective Sine-Cosine Algorithm (MOSCA)^49^, based on the search mechanism of the sine-cosine algorithm (SCA)^50^, was proposed by Tawhid and Savsani. MOSCA employs elite non-dominated ranking and crowded distance techniques to preserve diversity and distinguish various non-dominated levels within the Pareto optimal solution sets. Recent advancements in metaheuristic algorithms have shown the benefit of hybrid and adaptive strategies to enhance optimization performance. Widians et al. proposed a hybrid Ant Colony Optimization and Grey Wolf Optimizer algorithm that effectively balances exploration and exploitation, leveraging the complementary strengths of ACO’s global search and GWO’s exploitation capabilities, leading to improved convergence in complex optimization landscapes^51^. Similarly, Farda et al. introduced an Adaptive Differential Evolution algorithm featuring multiple crossover strategies that dynamically adjust mutation and crossover parameters according to the evolutionary stage, which enhances the balance between diversity and convergence efficiency across diverse problems^52^. Additionally, Hedayati-Dezfooli et al. applied soft computing techniques combined with fuzzy evaluation and the Taguchi method to optimize injection molding processes for aviation propellers, exemplifying the integration of multi-objective optimization with domain-specific manufacturing challenges^53^. These studies collectively highlight the continued evolution of multi-objective metaheuristics through hybridization, adaptive parameter control, and application-driven optimization.

Other notable algorithms include the Multi-objective Chimp Optimization Algorithm (MoChOA)^42^, Multi-objective Grasshopper optimization (MOGOA)^54^, Multi-objective Seagull Optimization Algorithm^14^, Multi-objective marine predator algorithm (MOMPA)^55^, Multi-objective Sparrow search Algorithm (MOSSO^56^, Multi-objective Ant-lion Optimizer^57^, and the Multi-Objective Graylag Goose optimization^58^ are also recognized for their contributions to the field. These algorithms offer various innovative approaches to solving complex multi-objective optimization challenges. A new algorithm is often designed to address problems that existing ones cannot solve. In the following section, advanced multi-objective version of the recently developed HEOA is presented to effectively find optimal solutions for multi-objective problems.

## Fundamental human evolutionary optimization algorithm

This section provides a detailed discussion on the inspiration and mathematical modeling of the proposed algorithm.

### Evolutionary framework

HEOA is based on how human evolution adapts to find the best solutions in difficult situations. This process of survival and change helped create HEOA, a method for solving complex problems efficiently. It is also inspired by the Chaotic Universe Theory^59^, which says the universe evolves through chaotic, self-organizing processes. HEOA uses a technique called Logistic Chaos Mapping^60^ to start the process. By including chaos theory, HEOA adds a flexible and exploring approach to solving problems. Human evolution can be divided into two phases: exploration and development. The exploration phase involved early humans using trial and error to adapt to new environments, building foundational knowledge and skills. This phase reflects humanity’s initial quest for survival and organization. The development phase saw the rise of complex societies, driven by knowledge accumulation, observation, and adaptation. Humans created systems for communication, trade, and governance, advancing in arts, sciences, and philosophies. HEOA categorizes society into four roles:

1. Leaders guide progress using existing knowledge.
2. Seekers explore new opportunities.
3. Followers adopt leaders’ methods.
4. Losers, unable to adapt, are phased out.

Each role uses different strategies: leaders build on knowledge, seekers explore, followers follow, and losers fall behind. This framework highlights the balance between progress and adaptation in human evolution.

### Initialization

To model the chaotic phase at the beginning of human evolution, the HEOA utilizes Logistic Chaos Mapping to initialize the population. The initialization process, based on Logistic Chaos Mapping, is defined as

3 $$\begin{aligned} x_{i} = \alpha \cdot x_{i-1}\cdot (1 - x_{i-1}) \quad 0 \le x_0 \le 1, \quad i = 1, 2, \dots , N, ~~ \alpha = 4 \end{aligned}$$

where $x_{i}$ denotes the value at the $i$-th iteration, and $x_{i-1}$ corresponds to the value from the previous iteration with maximum number of iteration defined as ${I_{max}}$. The chaotic value, $x_{i}$, is then mapped to the search space:

4 $$\begin{aligned} x_{i}^{0} = lb + (ub - lb) \end{aligned}$$

where *N* is the population size, $\text {Maxiter}$ represents the maximum number of iterations, and *lb* and *ub* are the lower and upper bounds of the search space, respectively.

### Human exploration strategy

Once the population is initialized, the subsequent task is to evaluate the fitness of each solution. The exploration phase was defined as the first quarter of the maximum number of iterations. Throughout human history, individuals typically rely on a uniform search strategy when venturing into unknown territories with limited understanding. This behavior can be mathematically expressed by the following formula, represented as:

5 $$\begin{aligned} X_{i}^{{t + 1}} & = \beta \left( {1 - \frac{t}{{I_{{max}} }}} \right)(X_{i}^{t} - X_{{{\mathrm{best}}}} )Levy(dim) + X_{{{\mathrm{best}}}} \left( {1 - \frac{t}{{I_{{max}} }}} \right) \\ & \quad + (X_{{{\mathrm{mean}}}}^{t} - X_{{{\mathrm{best}}}} ) \cdot floor\left( {\frac{{rand}}{{f_{{jump}} }}} \right) \cdot f_{{jump}} \\ \end{aligned}$$

where *t* is the current iteration, *dim* is the problem’s dimensionality, and $X_i^t$ and $X_i^{t+1}$ represent the current and updated positions, respectively. $X_{\text {best}}$ is the best position found so far, and $X_{\text {mean}}^t$ is the average position of the population. The $\text {floor}$ function rounds down, *Levy* denotes the *Levy* distribution, $f_{\text {jump}}$ is the jump coefficient, and $\text {rand}$ is a random number in [0, 1]. All these parameters are thoroughly explained by^8^.

### Human growth phase

According to the HEOA, human progress can be divided into four key societal roles: leaders, explorers, followers, and losers. Each group adopts a unique method for seeking solutions, and their combined efforts contribute to finding the best global outcome. Each role has its own defined search strategy, as outlined in the following section:

1. In optimization algorithms, leaders are the smartest solutions, usually found in the best areas of the search space. For the experiment, the top 40% of solutions with the highest initial scores are called leaders. These leaders initiate a search for more optimal regions within the solution space, utilizing their existing knowledge to improve the overall performance and guide the exploration process towards more promising areas for optimization.
  6 $$\begin{aligned} X_i^{t+1} = {\left\{ \begin{array}{ll} \gamma \cdot X_i^t ~ \exp \left( \frac{-t}{rand . I_{max}}\right) , & \text {if } rand < A, \\ \gamma \cdot X_i^t + randn \cdot {ones}(1, {dim}), & \text {if } rand \ge A. \end{array}\right. } \end{aligned}$$
  The term *randn* refers to a random number drawn from a standard normal distribution. The function *ones(1, dim)* creates a row vector with *dim* elements, all set to 1. Meanwhile, *rand* generates a uniformly random value between 0 and 1, representing the complexity of the leader’s current situation. The variable quantifies the situation’s evaluation value, which was experimentally set to 0.6. Based on the complexity, the leader selects an appropriate search strategy. The coefficient $\gamma$, representing the ease of knowledge acquisition, decreases progressively as development advances, given as:
  7 $$\begin{aligned} \gamma = 0.2 \cos \left( \frac{\pi }{2} (1-\frac{t}{I_{max}})\right) \end{aligned}$$
2. Explorers are essential for navigating unexplored areas in search of the global optimum. In the experiment, individuals with fitness levels ranking between the top 40% and 80% of the population are categorized as explorers. Their behavior during the search process is described by the following strategy:
  8 $$\begin{aligned} X_i^{t+1} = randn \cdot \exp \left( \frac{X_{\text {worst}}^{t^2} - X_i^{t^2}}{i^2}\right) \end{aligned}$$
  $X_{worst}^{t}$represents the position vector of the least fit individual in the population at iteration *t*.
3. Followers align themselves with the direction set by the most adaptable leader, mimicking their behavior. Specifically, those who rank in the top 80% to 90% based on their adaptability assume the role of followers. The approach used by these followers in their search can be formulated as follows:
  9 $$\begin{aligned} X_i^{t+1} = X_i^{t} + \gamma \cdot rand (X_{{best}}^{t}- X_{i}^{t}) \end{aligned}$$
  $X_{{best}}^{t}$ denotes the position of the most highly adapted individual in the human population at the $t^{th}$ iteration.
4. Individuals exhibiting low levels of adaptation are deemed unsuccessful and are systematically removed from the population due to their inability to integrate effectively within the societal framework. To sustain a stable population size, new individuals are introduced through reproduction in regions conducive to human development. This regeneration process is governed by the following formulation
  10 $$\begin{aligned} X_i^{t+1} = X_{best} + (X_{best} - X_{i}^{t}). randn \end{aligned}$$

## Proposed multi-objective human evolutionary optimization algorithm (MOHEOA)

### Motivation

**Fig. 2:**
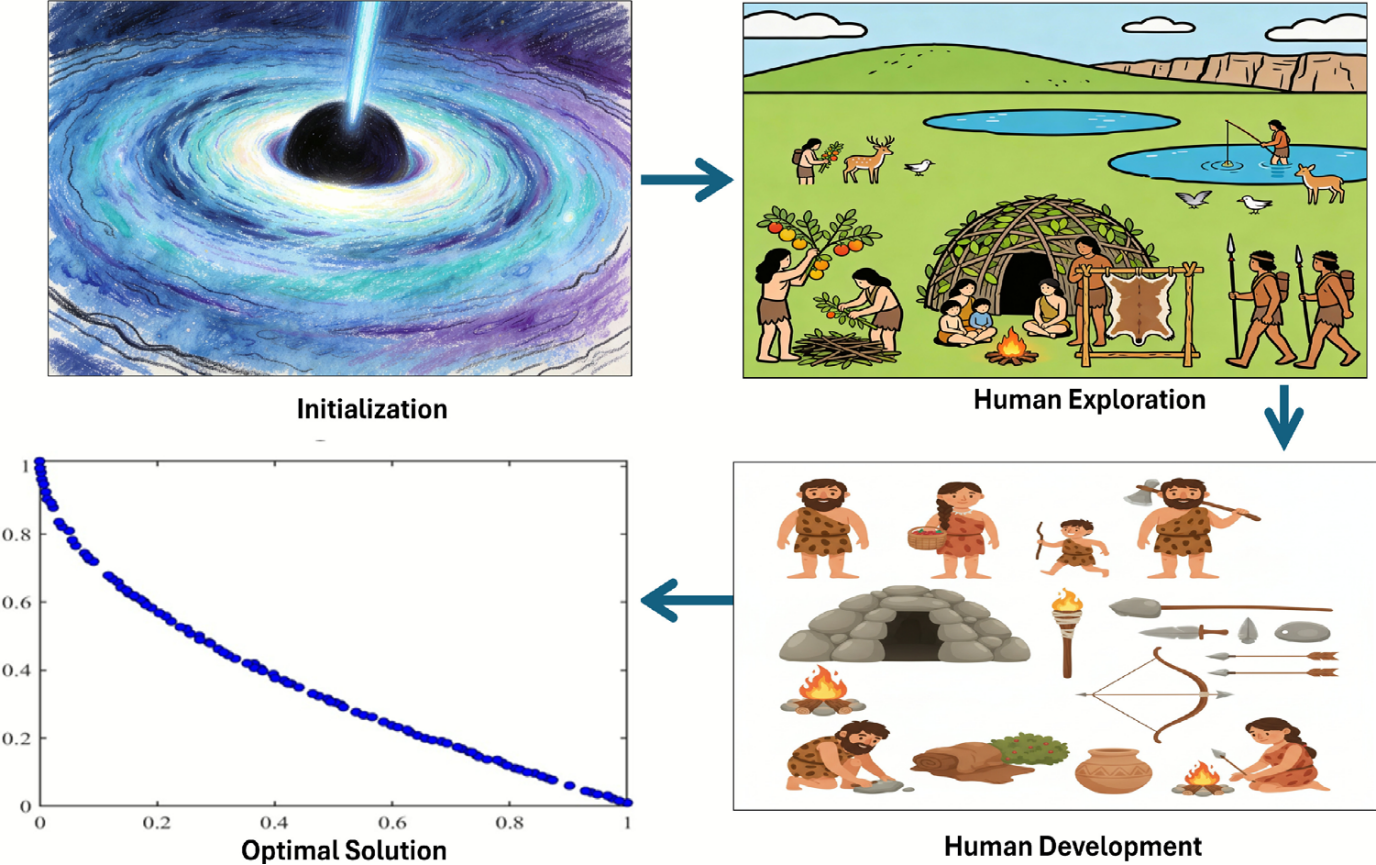
Framework and mechanism of the proposed Multi-Objective Human Evolutionary Optimization Algorithm (MOHEOA).

In the process of solving optimization problems using metaheuristic approaches, achieving an optimal balance between exploration and exploitation is crucial for the algorithm to identify the most effective solutions. These algorithms are favored by researchers due to their simple design, ease of implementation, and the fact that they do not require derivatives. However, a significant challenge these algorithms face is the tendency for control parameters to change during their execution. Another limitation is that these algorithms may fail to converge to the global optimum, often becoming trapped in sub-optimal solutions during the search process. The exploration phase drives diversity by conducting a global search to generate new solutions, while the exploitation phase focuses on refining these solutions by exploring local neighborhoods , ensuring that convergence to the optimum is achieved^48^. The underlying principle of the MOHEOA algorithm is inspired by the process of human evolution, integrating both historical and social aspects of human development. The pictorial representation of proposed MOHEOA is given in Fig. 2. To adapt a single-objective HEOA for multi-objective HEOA, two additional components are introduced, drawing inspiration from MOPSO^38^. The first component is the archive, which serves as a repository for the Pareto optimal solutions identified throughout the optimization process. The second component is the selection mechanism, responsible for selecting the most optimal solution from the archive. The psudeocode of proposed MOHEOA is given as Algorithm 1.

**Algorithm 1:**
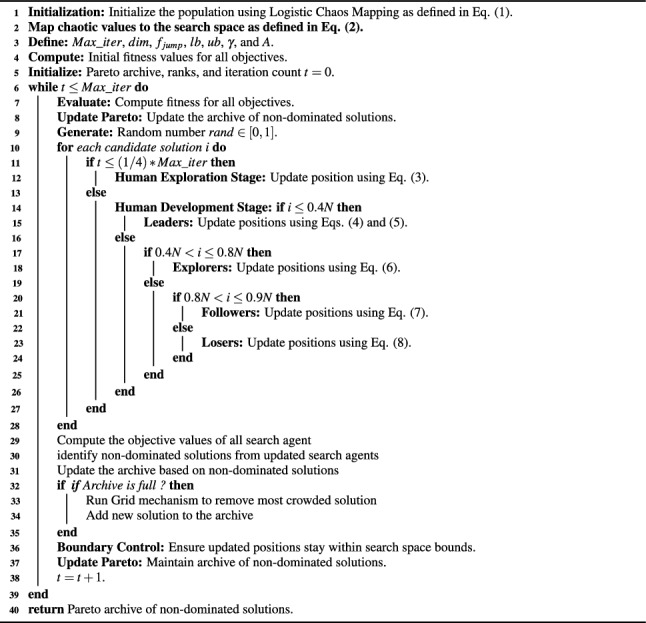
Multi-Objective Human Evolutionary Algorithm (MOHEA).

### Archive

The archive is employed to store the non-dominated optimal solutions, maintaining a collection of the Pareto-optimal solutions identified throughout the optimization process. The archive typically has a fixed capacity, often set to half the population size. It systematically accumulates solutions from the current population and updates itself by performing a comparison between the newly generated solutions and those already stored. The update process is carried out as follows:

- If the newly generated solution from a population update is dominated by any solution in the archive, it will not be added to the archive.
- If the new solution from the population update dominates one or more solutions in the archive, the dominated solutions will be removed, and the new solution will replace them.
- The new solution will be added to the archive as a new member only if no existing solution in the archive dominates it.

**Fig. 3:**
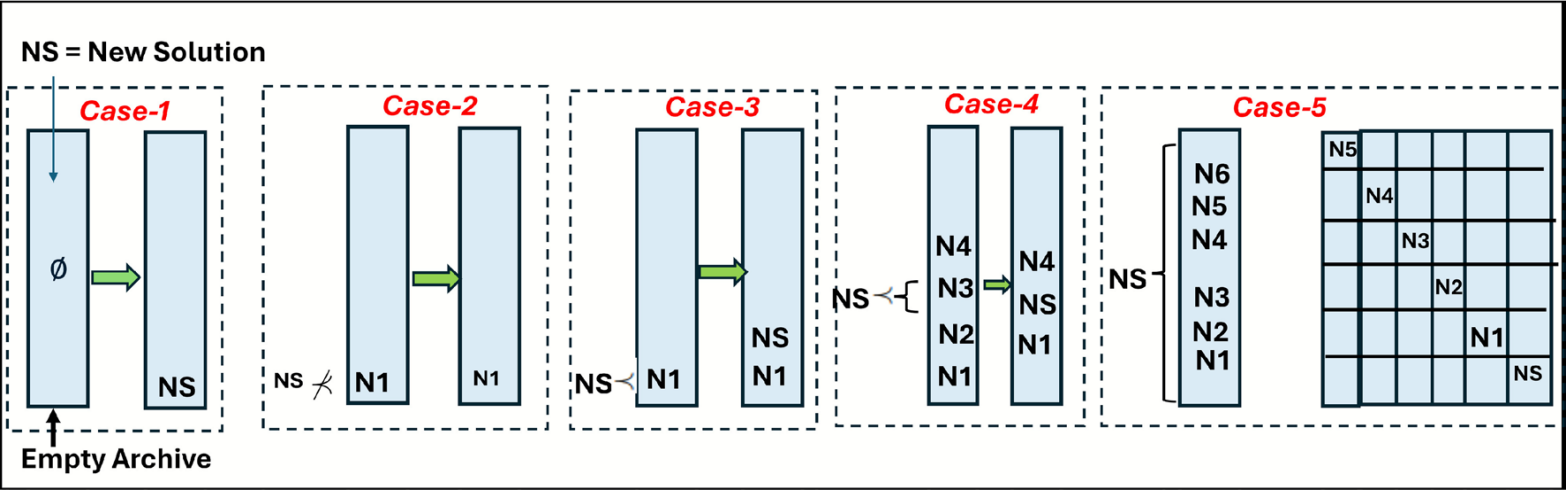
Different cases for insertion of new solution in Archive controller.

Based on the archiving update criteria, all solutions stored in the archive dominate the remaining solutions in the population. A specific case requires careful consideration: when the archive has reached its maximum capacity and a new solution qualifies for inclusion, it cannot be added, which may result in the loss of a potentially optimal solution. To address this issue, one commonly used approach is to remove an existing solution at random. However, this removal should be done thoughtfully, with attention to the overall distribution of solutions in the archive. Ideally, the solution selected for removal should have the least impact on the diversity of the archive. To evaluate the distribution, a niche-based method is applied. In this approach, each solution is assigned a fixed radius, and the number of neighboring solutions within this radius is counted. This count serves as a crowding metric. The distance used for this calculation is defined as follows:

11 $$\begin{aligned} \vec {d} = \frac{S_{max}-S_{min}}{sizeof(Archieve)} \end{aligned}$$

here, $S_{\text {max}}$ and $S_{\text {min}}$ are vectors that contain the maximum and minimum values for each objective, respectively, while $\text {sizeof(Archive)}$ represents the total number of solutions stored in the archive. Fig. 3 illustrates how the archive controller updates solutions. Initially, the archive is empty, and the first solution is stored directly (Fig. 3, Case-1). When a new solution is produced, it is evaluated against the archive’s current solutions. If the new solution is inferior (dominated) or identical to an existing one, it is rejected (Fig. 3, Case-2). However, if the new solution outperforms an archived solution or is non-dominated, it is added to the archive (Fig. 3, Case-3 and Case-4). If the archive reach capacity, a grid-based method divides the objective space into segments (hypercubes), and the most densely populated segment is trimmed to accommodate the new solution in a less crowded area (Fig. 3, Case-5).

### Grid mechanism

**Fig. 4:**
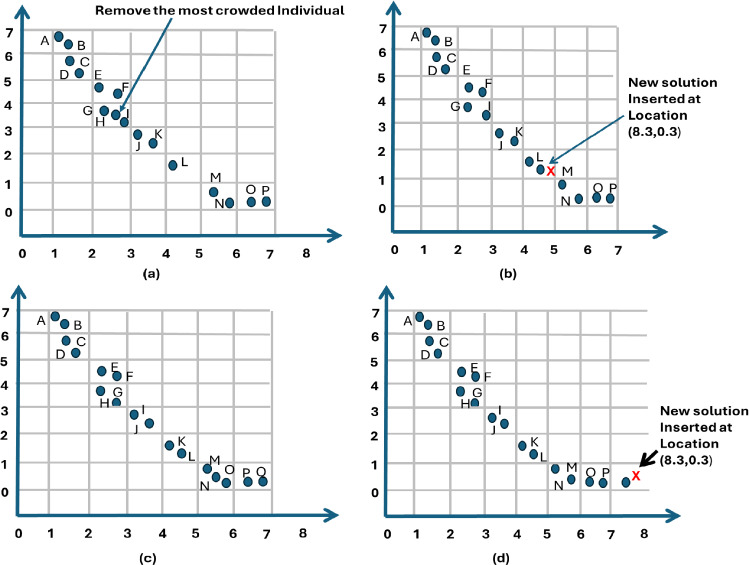
Pictorial representation of Grid operation (**a**) Division of objective space into different grids. (**b**) Insertion in adaptive grid when element lies within the current boundaries of the grid. (**c**) identification of crowded region. (**d**) Repositioning the solutions if the newly added solution falls beyond the grid’s boundary.

The adaptive grid method is utilized to generate distributions of Pareto fronts^61^. The concept revolves around utilizing an external archive to store all non-dominated solutions along with their corresponding information. Within the archive, the objective space is systematically divided into multiple regions, as depicted in Fig.4a. The likelihood of losing a solution increases proportionally with the density of solutions within a specific hypercube or subdivision. When the archive reaches its maximum capacity, some solutions are removed to make room for new ones. Specifically, a solution is selected for deletion from the most densely populated region of the archive, ensuring space for the incoming solution while maintaining balance as Fig. 4b. In another case, If a newly added individual falls outside the existing boundaries of the grid, the grid must be redefined, and all individuals need to be repositioned accordingly^48,62^ as depicted in Fig. 4d. The adaptive grid is composed of hypercubes, serving as a framework to ensure a uniform distribution across the space.

### Leader selection

In multi-objective optimization, a key challenge is to effectively compare new candidate solutions with the existing solutions in the search space. This comparison is critical to maintaining a diverse and well-distributed Pareto front. One approach to address this challenge is through a *leader selection* method that biases selection towards regions of the search space that are less crowded. Specifically, the search space is divided into multiple segments or grids, and the selection process favors solutions located in the sparsely populated segments to enhance diversity. This is achieved by assigning each segment $k$ a selection weight $U_{k}$ defined as:

12 $$\begin{aligned} U_{k} = \frac{g}{N_{k}}, \end{aligned}$$

where $g > 1$ is a constant that controls the selection pressure, and $N_k$ denotes the number of Pareto optimal solutions currently present in the $k$-th segment. The inverse relationship implies that segments with fewer solutions are assigned higher selection weights.

To elaborate with a mini-example: suppose the archive is divided into 3 segments (k=1,2,3) containing $N_1 = 5$, $N_2 = 1$, and $N_3 = 2$ solutions, respectively. Using $g = 2$, the selection weights are calculated as $U_1 = 2/5 = 0.4$, $U_2 = 2/1 = 2.0$, and $U_3 = 2/2 = 1.0$. These weights are normalized to form a probability distribution for a roulette wheel. The total weight is $0.4 + 2.0 + 1.0 = 3.4$. The probability of selecting a segment is then $P_1 = 0.4/3.4 \approx 0.12$, $P_2 = 2.0/3.4 \approx 0.59$, and $P_3 = 1.0/3.4 \approx 0.29$. A random number between 0 and 1 is generated to spin the wheel. If the number falls in [0.00, 0.12), segment 1 is chosen; if in [0.12, 0.71), segment 2; and if in [0.71, 1.00), segment 3. Despite having only one solution, segment 2 occupies about 59% of the wheel due to its sparsity.

**Fig. 5:**
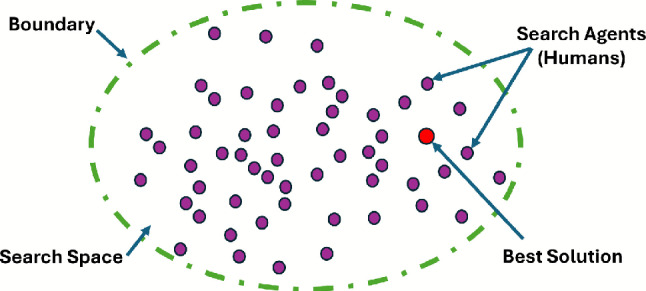
Selection of leader.

These probabilities are used to construct the roulette wheel. A random number is then generated to simulate a spin, and the segment corresponding to the random number’s range is selected. From that segment, a leader solution is chosen uniformly at random. This probabilistic strategy ensures that leaders are preferentially selected from less crowded regions, which promotes exploration and preserves diversity across the Pareto front. Figure 5 illustrates this concept, highlighting how solutions occupy different proportions of the roulette wheel based on their segment density, enabling a balanced trade-off between convergence and diversity in the multi-objective search process.

### Computational complexity

This section presents a detailed analysis of the computational efficiency of the proposed method.

#### Time complexity

The initialization of the MOHEOA population requires a computational complexity of $O(n_o \times n_p)$, where $n_o$ denotes the number of objectives and $n_p$ represents the population size. The fitness calculation for each search agent incurs a complexity of $O$($I_{max} \times n_o \times n_p)$, where $I_{max}$ is the maximum number of iterations used to simulate the proposed MOHEOA algorithm. Furthermore, defining the group of the human population has a time complexity of $O(N)$, where $N$ represents the total count of individuals in the population $n_p$. Updating the archive of non-dominated solutions requires $O(n_o \times (n_{\text {ns}} + n_p))$, where $n_{\text {ns}}$ refers to the number of non-dominated solutions. Therefore, the overall time complexity of the proposed MOSHO algorithm is given by:

13 $$\begin{aligned} O(I_{max} \times n_o \times (n_p + n_{\text {ns}})) \end{aligned}$$

#### Space complexity

The space complexity of the MOHEOA algorithm is analyzed during the initialization phase, where memory is allocated for the process at any given time. Consequently, the overall space complexity of the MOHEOA algorithm is:

14 $$\begin{aligned} O(n_o \times n_p) \end{aligned}$$

#### Comparative analysis of time complexity

A comparative analysis of computational complexity places the proposed MOHEOA within the context of other prominent multi-objective optimizers. The time complexity of MOHEOA, $O(I_{\text {max}} \times n_o \times (n_p + n_{\text {ns}}))$, is comparable to that of well-established algorithms. For instance, the NSGA-II algorithm has a complexity of $O(I_{\text {max}} \times n_o \times n_p^2)$, which is dominated by its non-dominated sorting and crowding distance calculation procedures^63^. In contrast, the MOEA/D algorithm exhibits a lower complexity of $O(I_{\text {max}} \times n_o \times n_p \times T)$, where $T$ is the neighborhood size and is typically much smaller than the population size $n_p$^41^. Consequently, the complexity of MOHEOA lies between the more efficient MOEA/D and the more computationally intensive NSGA-II. The archive management mechanism in MOHEOA is not excessively costly; in fact, it can be more efficient than the sorting approach of NSGA-II for larger populations, as the term $(n_p + n_{\text {ns}})$ is typically more favorable than $n_p^2$. The added computational expense compared to MOEA/D is justified by the explicit maintenance of a diverse Pareto-optimal set, a key strength of archive-based approaches that enhances performance on problems with complex Pareto front geometries.

## Experimental result and discussion

### Experimental setup

The experiments were conducted using MATLAB 2025 (a) on a system equipped with an Apple M1 chip, 8-core GPU, 8GB memory, and 512GB storage. The proposed algorithm, along with other comparative approaches, was implemented and tested under the same computational environment to ensure consistency and fairness in performance evaluation.

### Benchmark test functions

This section evaluates the performance of the proposed MOHEOA using 23 unconstrained multi-objective benchmark functions and , which are categorized as follows.

1. Unconstrained benchmark function: The proposed MO-HEOA is tested on various types of unconstrained optimization problems. The First is ZDT family of functions *(ZDT1-ZDT4, ZDT6)*^13^, widely used for benchmarking multi-objective Pareto optimization methods^64–66^. The proposed method is also tested on continuous MOPs with complex Pareto sets, drawn from CEC 2009 competition on multi-objective evolutionary algorithms^35^. The problems, categorized as *UF1–UF10*, include *UF1–UF7* with two objectives and *UF8–UF10* with three objectives, providing a comprehensive assessment of algorithm performance. The proposed model is also tested on recently developed IMOP test problems^36^, which is one of the most challenging problem for multi-objective optimization. These test suites include MOPs that have irregular PF shapes to test the capability of a MOEA to generate well-diversified approximations sets. It addresses challenges such as separability, multi-modality, and varied PF geometries, including concavity, convexity, and discontinuities.
2. Constrained benchmark function: To further validate its robustness, the proposed MOHEOA algorithm was rigorously tested on four challenging constrained engineering design problems: (1) Welded Beam Design—minimizing cost while satisfying shear stress and deflection constraints, (2) Multiple-Disk Clutch Brake Design—optimizing mass and stopping time under thermal and geometric constraints, (3) Gear Train Design—achieving optimal gear ratio with manufacturing limitations, and (4) 25-Bar Truss Structure—minimizing weight while meeting nodal displacement and stress requirements.

### Performance metrics and ranking

To evaluate the performance of a multi-objective evolutionary algorithm, three major criteria are used^13,62^: (1) Minimizing the distance between the non-dominated set generated by the algorithm and the Pareto Front (PF) of the multi-objective problem (MOP), measured using the *Delta-p (*$\Delta _p$*)* metric^67^; (2) Maximizing the diversity and uniform distribution of solutions along the PF, quantified via the *Spread* metric^68^; and (3) Maximizing the coverage of the PF by the non-dominated solutions, assessed through the *Hypervolume (HV)*^69^ and *Epsilon (*$\epsilon$*)*^68^ indicators. For these criteria, four widely used performance metrics are employed to measure the efficiency of the proposed MOHEOA^70,71^.

### Hypervolume (HV)

It measures the volume of the objective space dominated by the approximate Pareto front $P$ relative to a reference point $\textbf{r}$.

15 $$\begin{aligned} HV(P, \textbf{r}) = \text {Volume}\left( \bigcup _{\textbf{y} \in P} \left[ y_1, r_1 \right] \times \left[ y_2, r_2 \right] \times \dots \times \left[ y_m, r_m \right] \right) \end{aligned}$$

where $P$ is Approximate Pareto front (set of non-dominated solutions), $\textbf{r} = (r_1, r_2, \dots , r_m)$ is Reference point (must be dominated by all solutions in $P$). $m$ is Number of objectives. Higher HV shows the better convergence and diversity.

### $\Delta _p$ (Delta-p, $p = 1$)

It measures the average distance between the obtained Pareto front $P$ and a reference front $P^*$.

16 $$\begin{aligned} \Delta _1(P, P^*) = \frac{1}{|P^*|} \sum _{\textbf{y}^* \in P^*} \min _{\textbf{y} \in P} d(\textbf{y}, \textbf{y}^*) \end{aligned}$$

where: $d(\textbf{y}, \textbf{y}^*)$ is the Euclidean distance between solutions $\textbf{y}$ and $\textbf{y}^*$ and $P^*$ is the Reference Pareto front. Lower value of $\Delta _1$ shows the better convergence.

### Spread (Diversity Metric)

Spread metrics evaluates the uniformity of solution distribution along the Pareto front. Mathematically, it is given as

17 $$\begin{aligned} \text {Spread}(P) = \frac{d_f + d_l + \sum _{i=1}^{|P|-1} |d_i - \bar{d}|}{d_f + d_l + (|P| - 1)\bar{d}} \end{aligned}$$

where, $d_i$ is the distance between consecutive solutions in $P$, $\bar{d}$ is the average of $d_i$ and $d_f, d_l$ is the distances to the extreme solutions of the true Pareto front. Lower spread value shows more uniform distribution.

### Epsilon ($\epsilon$) Indicator

Epsilon ($\epsilon$) Indicator measures the smallest value $\epsilon$ needed so that one Pareto front becomes weakly dominated by another.

18 $$\begin{aligned} I_{\epsilon +}(P, P^*) = \inf _{\epsilon \in \mathbb {R}} \left\{ \forall \textbf{y}^* \in P^*, \exists \textbf{y} \in P : y_i \le y^*_i + \epsilon \text { for all } i \right\} \end{aligned}$$

where, $P$ is the approximate Pareto front and $P^*$ represent the Reference Pareto front. Lower $I_{\epsilon +}$ values show a better approximation of the obtained Pareto front.

#### Experimental setting

To evaluate the effectiveness of the MOHEOA algorithm, its performance is benchmarked against six established optimization techniques: MOPSO, NSGA-2, SPEA-2, MOEA/D, MOGWO, and MOChOA. The selection behind these algorithms is based on their established reputation as state-of-the-art methods, ensuring a fair comparison against widely accepted benchmarks. These algorithms represent diverse optimization paradigms, including swarm intelligence, evolutionary algorithms, and decomposition-based approaches, providing a comprehensive evaluation across different problem-solving strategies. The parameter settings for these algorithms are implemented according to the recommendations provided by their developers in original publications.

### Simulation results for benchmark functions

To assess the performance of proposed MOHEOA algorithm, A comparative analysis is conducted against several established algorithms, including MOPSO, NSGA-2, SPEA-2, MOEA/D, MOGWO, and the recently introduced MOChOA. This evaluation was performed across all 23 test problems, utilizing performance metrics such convergence metrics, DM, GD, IGD and HVD. The initial population size was configured at 100 for the experiments. The maximum function evaluation for bi-objective and tri-objective functions is kept 30000 and 50000, respectively. Thus, the maximum number of iterations is determined as $\frac{25,000}{100} = 250$ for bi-objective benchmark functions and $\frac{50,000}{100} = 500$ for tri-objective benchmark functions. The performance evaluation of the proposed MOHEOA and the comparative algorithms, NSGA-II, MOChOA, MOPSO, MOEA/D, and SPEA2, was conducted using our MATLAB implementation, which is freely accessible on the MathWorks website. The default parameter configurations for NSGA-II, MOChOA, MOPSO, MOEA/D, and SPEA2 are provided in Table 1.

**Table 1:** Parameter settings of MOPSO, NSGA-II, MOEA/D, SPEA2, MOGWO, and MOChOA.

No.	MOPSO	NSGA-II	MOEA/D	SPEA2	MOGWO	MOChOA
1	$\phi _a = \phi _b = 2.05$	Population size (*N*) = 100	Subproblems (*N*) = 100	Population size (*N*) = 100	Grid inflation ($\alpha$) = 0.1	–
2	Inertia weight: $w = \dfrac{2}{\phi _f - 2 + \sqrt{\phi _f^2 - 4\phi _f}}$	Crossover prob. ($P_c$) = 0.8	Neighbors (*T*) = 0.1*N*	Crossover prob. ($P_c$) = 0.8	Leader pressure = 2	Random vectors $r_1, r_2 = 0.1$
3	Personal coeff. $c_1 = x \times \phi _a$	Mutation prob. ($P_m$) = 0.1	Max copies (*M*) = 0.01*N*	SBX index = 15	Grids/dim. = 10	Chaotic vectors (*m*)
4	Social coeff. $c_2 = x \times \phi _b$	–	Parent selection ($P_p$) = 0.9	Mutation prob. ($P_m$) = 1/*n*	–	Nonlinear coeff. (*f*)
5	Grid inflation ($\alpha$) = 0.1	–	Mutation rate (*Mr*) = 0.5	Mutation index = 20	–	–
6	Leader pressure ($\beta$) = 4	–	Distribution index ($D_i$) = 30	–	–	–
7	Grid count = 10	–	–	–	–	–

Further details can be found in the respective original literature. All algorithms were executed independently 30 $\times$, and all four performance metrics were computed for each run. First, the proposed MOHEOA is analyzed using the HV metric. HV metric is an important indicator used to evaluate the performance of the proposed MOHEOA compared to other algorithms. The results are depicted in Table 2. MOHEOA consistently achieves the highest mean HV values across most test functions, demonstrating superior performance in multi-objective optimization. In the ZDT problems, MOHEOA outperforms all other algorithms in ZDT1, ZDT2, ZDT3, and ZDT4, achieving the highest HV values. Even in ZDT6, it remains highly competitive with a value of 3.23E-01, surpassing NSGA-II, MOChOA, and MOEAD. Similarly, in the IMOP problems, MOHEOA dominates in IMOP1, IMOP2, IMOP3, IMOP4 (2.42E-01), and IMOP6, consistently achieving the best HV values. This superior performance highlights its strong convergence and diversity in multi-objective optimization. For the UF problems, MOHEOA continues to excel, obtaining the highest mean HV values in UF1, UF2, UF3, UF4, UF5, and UF6. Additionally, its standard deviation values are either lower or comparable to other algorithms, indicating greater stability and robustness. While certain algorithms, such as SPEA2, perform well in specific cases, MOHEOA consistently maintains its dominance, making it the best-performing algorithm in terms of the HV metric. The results obtained using $\Delta _{p}$ are depicted in the Table 3. It is observed from Table 3, MOHEOA outperforms the other algorithms, achieving the best results in 21 out of the 23 test problems. The best results in terms of mean and standard deviation *(STD)* are highlighted in bold in the Table 3. Following MOHEOA, NSGA-II algorithm also performs well, and the results are close to the MOHEOA. SPEA2 algorithm shows the weakest performance among all algorithms in terms of $\Delta _{p}$ metric. Table 4 demonstrates that MOHEOA achieves the best results in most test functions in terms of Spread metrics. In multi-objective optimization, a lower spread value indicates better diversity and uniformity of solutions along the Pareto front. Across multiple benchmark problems, MOHEOA consistently achieves the lowest mean spread values, signifying its ability to maintain a well-distributed Pareto front. For test functions such as ZDT1, ZDT2, ZDT3, ZDT4, and ZDT6, MOHEOA significantly outperforms the other algorithms, achieving the lowest spread values, which confirms its superior diversity preservation. Similarly, in the IMOP series, MOHEOA achieves smaller spread values, particularly in IMOP1, IMOP2, IMOP3, and IMOP5, further establishing its effectiveness in maintaining uniform solutions. Additionally, for the UF problem set, MOHEOA delivers the best spread results in multiple cases, including UF1, UF2, UF3, UF4, and UF5, reaffirming its robustness across different optimization scenarios. MOHEOA not only achieves lower mean spread values but also maintains smaller standard deviations, indicating stable and consistent performance across independent runs. Although NSGA-II and MOChOA perform well in specific instances, their performance varies significantly across different test problems.

**Table 2:** Performance comparison using Hypervolume (HV) metric across benchmark problems, with bold values indicating best results.

Test problem	M	D	MOHEOA		NSGA-II		MOChOA		MOPSO		MOEA/D		SPEA2	
			Mean	SD	Mean	SD	Mean	SD	Mean	SD	Mean	SD	Mean	SD
ZDT														
ZDT1	2	30	**7.06**$\times 10^{-1}$	**2.79**$\times 10^{-3}$	1.51$\times 10^{-1}$	7.07$\times 10^{-2}$	5.58$\times$ $10^{-1}$	6.84$\times$ $10^{-2}$	7.02$\times$ $10^{-1}$	3.71$\times$ $10^{-3}$	6.97$\times$ $10^{-1}$	4.33$\times$ $10^{-3}$	5.07$\times$ $10^{-1}$	1.51$\times$ $10^{-2}$
ZDT2	2	30	**4.13**$\times$ $10^{-1}$	**2.68**$\times$ $10^{-2}$	1.51$\times$ $10^{-3}$	8.25$\times$ $10^{-3}$	1.05$\times$ $10^{-1}$	2.73$\times$ $10^{-2}$	3.96$\times$ $10^{-1}$	7.27$\times$ $10^{-2}$	3.97$\times$ $10^{-1}$	5.31$\times$ $10^{-2}$	2.68$\times$ $10^{-1}$	1.88$\times$ $10^{-3}$
ZDT3	2	30	5.91$\times$ $10^{-1}$	1.96$\times$ $10^{-2}$	2.98$\times$ $10^{-1}$	8.94$\times$ $10^{-2}$	5.60$\times$ $10^{-1}$	4.70$\times$ $10^{-2}$	5.96$\times$ $10^{-1}$	1.65$\times$ $10^{-2}$	5.95$\times$ $10^{-1}$	2.24$\times$ $10^{-2}$	**6.95**$\times$ $10^{-1}$	**1.81**$\times$ $10^{-2}$
ZDT4	2	10	**5.09**$\times$ $10^{-1}$	**1.24**$\times$ $10^{-1}$	0.00$\times$ $10^{0}$	0.00$\times$ $10^{0}$	1.95$\times$ $10^{-1}$	1.55$\times$ $10^{-1}$	4.83$\times$ $10^{-1}$	1.30$\times$ $10^{-1}$	2.87$\times$ $10^{-1}$	1.77$\times$ $10^{-1}$	1.52$\times$ $10^{-1}$	1.06$\times$ $10^{-1}$
ZDT6	2	10	**3.23**$\times$ $10^{-1}$	**3.00**$\times$ $10^{-2}$	2.25$\times$ $10^{-1}$	1.45$\times$ $10^{-1}$	2.83$\times$ $10^{-1}$	3.26$\times$ $10^{-2}$	3.23$\times$ $10^{-1}$	3.64$\times$ $10^{-2}$	2.01$\times$ $10^{-1}$	4.73$\times$ $10^{-2}$	2.23$\times$ $10^{-1}$	1.86$\times$ $10^{-2}$
IMOP														
IMOP1	2	10	**9.87**$\times$ $10^{-1}$	**2.25**$\times$ $10^{-3}$	5.21$\times$ $10^{-1}$	3.70$\times$ $10^{-1}$	9.66$\times$ $10^{-1}$	1.95$\times$ $10^{-3}$	9.81$\times$ $10^{-1}$	1.70$\times$ $10^{-2}$	9.59$\times$ $10^{-1}$	2.19$\times$ $10^{-2}$	4.00$\times$ $10^{-1}$	2.65$\times$ $10^{-1}$
IMOP2	2	10	**1.07**$\times$ $10^{-1}$	**3.31**$\times$ $10^{-2}$	9.63$\times$ $10^{-2}$	3.20$\times$ $10^{-2}$	9.09$\times$ $10^{-2}$	4.06$\times$ $10^{-7}$	9.38$\times$ $10^{-2}$	3.06$\times$ $10^{-3}$	9.54$\times$ $10^{-2}$	8.08$\times$ $10^{-3}$	9.10$\times$ $10^{-2}$	2.06$\times$ $10^{-2}$
IMOP3	2	10	4.19$\times$ $10^{-1}$	1.04$\times$ $10^{-1}$	5.10$\times$ $10^{-1}$	1.95$\times$ $10^{-1}$	1.78$\times$ $10^{-1}$	6.97$\times$ $10^{-2}$	4.02$\times$ $10^{-1}$	1.23$\times$ $10^{-1}$	2.50$\times$ $10^{-1}$	2.41$\times$ $10^{-2}$	0.00$\times$ $10^{0}$	0.00$\times$ $10^{0}$
IMOP4	3	10	**2.42**$\times$ $10^{-1}$	**1.31**$\times$ $10^{-1}$	1.57$\times$ $10^{-1}$	1.45$\times$ $10^{-1}$	5.27$\times$ $10^{-2}$	3.34$\times$ $10^{-2}$	2.27$\times$ $10^{-1}$	1.14$\times$ $10^{-1}$	8.56$\times$ $10^{-2}$	7.09$\times$ $10^{-2}$	1.62$\times$ $10^{-1}$	9.46$\times$ $10^{-2}$
IMOP5	3	10	**5.20**$\times$ $10^{-1}$	**1.67**$\times$ $10^{-2}$	3.18$\times$ $10^{-1}$	8.14$\times$ $10^{-2}$	4.25$\times$ $10^{-1}$	3.01$\times$ $10^{-2}$	5.11$\times$ $10^{-1}$	1.29$\times$ $10^{-2}$	5.16$\times$ $10^{-1}$	1.73$\times$ $10^{-2}$	4.03$\times$ $10^{-1}$	1.16$\times$ $10^{-1}$
IMOP6	3	10	**4.85**$\times$ $10^{-1}$	**3.48**$\times$ $10^{-2}$	2.00$\times$ $10^{-1}$	1.21$\times$ $10^{-1}$	3.07$\times$ $10^{-1}$	1.92$\times$ $10^{-1}$	4.52$\times$ $10^{-1}$	1.28$\times$ $10^{-1}$	4.62$\times$ $10^{-1}$	1.02$\times$ $10^{-1}$	1.54$\times$ $10^{-2}$	3.67$\times$ $10^{-2}$
IMOP7	3	10	**9.58**$\times$ $10^{-2}$	**4.77**$\times$ $10^{-3}$	9.14$\times$ $10^{-2}$	9.79$\times$ $10^{-4}$	9.09$\times$ $10^{-2}$	1.07$\times$ $10^{-7}$	9.44$\times$ $10^{-2}$	1.13$\times$ $10^{-3}$	9.30$\times$ $10^{-2}$	2.02$\times$ $10^{-3}$	0.00$\times$ $10^{0}$	0.00$\times$ $10^{0}$
IMOP8	3	10	**5.20**$\times$ $10^{-1}$	**2.02**$\times$ $10^{-2}$	4.41$\times$ $10^{-1}$	4.77$\times$ $10^{-2}$	6.99$\times$ $10^{-2}$	1.62$\times$ $10^{-3}$	5.01$\times$ $10^{-1}$	3.34$\times$ $10^{-2}$	4.70$\times$ $10^{-1}$	2.02$\times$ $10^{-2}$	2.22$\times$ $10^{-1}$	1.18$\times$ $10^{-3}$
UF														
UF1	2	30	**5.69**$\times$ $10^{-1}$	**2.64**$\times$ $10^{-2}$	3.32$\times$ $10^{-1}$	5.77$\times$ $10^{-2}$	4.27$\times$ $10^{-1}$	5.91$\times$ $10^{-2}$	5.58$\times$ $10^{-1}$	3.74$\times$ $10^{-2}$	5.54$\times$ $10^{-1}$	4.48$\times$ $10^{-2}$	3.78$\times$ $10^{-1}$	6.72$\times$ $10^{-2}$
UF2	2	30	**6.47**$\times$ $10^{-1}$	**7.33**$\times$ $10^{-3}$	6.07$\times$ $10^{-1}$	1.17$\times$ $10^{-2}$	5.60$\times$ $10^{-1}$	2.36$\times$ $10^{-2}$	6.46$\times$ $10^{-1}$	6.12$\times$ $10^{-3}$	6.39$\times$ $10^{-1}$	5.37$\times$ $10^{-3}$	4.55$\times$ $10^{-1}$	1.81$\times$ $10^{-2}$
UF3	2	30	**3.24**$\times$ $10^{-1}$	**3.70**$\times$ $10^{-2}$	3.10$\times$ $10^{-1}$	4.51$\times$ $10^{-2}$	2.51$\times$ $10^{-1}$	4.39$\times$ $10^{-2}$	2.51$\times$ $10^{-1}$	4.41$\times$ $10^{-2}$	2.40$\times$ $10^{-1}$	3.92$\times$ $10^{-2}$	2.44$\times$ $10^{-1}$	1.98$\times$ $10^{-2}$
UF4	2	30	**3.45**$\times$ $10^{-1}$	**4.80**$\times$ $10^{-3}$	2.34$\times$ $10^{-1}$	1.47$\times$ $10^{-2}$	2.68$\times$ $10^{-1}$	7.30$\times$ $10^{-3}$	3.43$\times$ $10^{-1}$	4.69$\times$ $10^{-3}$	3.39$\times$ $10^{-1}$	3.75$\times$ $10^{-3}$	2.45$\times$ $10^{-1}$	1.76$\times$ $10^{-3}$
UF5	2	30	1.79$\times$ $10^{-2}$	2.90$\times$ $10^{-2}$	0.00$\times$ $10^{0}$	0.00$\times$ $10^{0}$	4.89$\times$ $10^{-4}$	2.68$\times$ $10^{-3}$	1.81$\times$ $10^{-2}$	3.26$\times$ $10^{-2}$	5.50$\times$ $10^{-2}$	6.25$\times$ $10^{-2}$	3.05$\times$ $10^{-2}$	3.88$\times$ $10^{-2}$
UF6	2	30	**1.31**$\times$ $10^{-1}$	**8.14**$\times$ $10^{-2}$	7.41$\times$ $10^{-3}$	1.53$\times$ $10^{-2}$	1.01$\times$ $10^{-1}$	6.99$\times$ $10^{-2}$	1.06$\times$ $10^{-1}$	7.88$\times$ $10^{-2}$	8.49$\times$ $10^{-2}$	5.61$\times$ $10^{-2}$	8.49$\times$ $10^{-2}$	6.39$\times$ $10^{-2}$
UF7	2	30	**3.94**$\times$ $10^{-1}$	**8.73**$\times$ $10^{-2}$	2.12$\times$ $10^{-1}$	7.25$\times$ $10^{-2}$	2.09$\times$ $10^{-1}$	7.37$\times$ $10^{-2}$	3.84$\times$ $10^{-1}$	8.44$\times$ $10^{-2}$	3.85$\times$ $10^{-1}$	8.15$\times$ $10^{-2}$	2.55$\times$ $10^{-1}$	4.47$\times$ $10^{-2}$
UF8	3	30	**3.01**$\times$ $10^{-1}$	**3.68**$\times$ $10^{-2}$	2.86$\times$ $10^{-1}$	1.71$\times$ $10^{-2}$	1.26$\times$ $10^{-1}$	3.90$\times$ $10^{-2}$	3.00$\times$ $10^{-1}$	1.55$\times$ $10^{-2}$	2.46$\times$ $10^{-1}$	4.66$\times$ $10^{-2}$	7.94$\times$ $10^{-2}$	2.02$\times$ $10^{-2}$
UF9	3	30	**3.54**$\times$ $10^{-1}$	**5.37**$\times$ $10^{-2}$	2.79$\times$ $10^{-1}$	3.77$\times$ $10^{-2}$	2.93$\times$ $10^{-1}$	5.56$\times$ $10^{-2}$	3.43$\times$ $10^{-1}$	5.14$\times$ $10^{-2}$	3.23$\times$ $10^{-1}$	5.61$\times$ $10^{-2}$	1.51$\times$ $10^{-1}$	2.35$\times$ $10^{-2}$
UF10	3	30	**5.35**$\times$ $10^{-2}$	**2.65**$\times$ $10^{-2}$	2.00$\times$ $10^{-2}$	2.24$\times$ $10^{-2}$	6.64$\times$ $10^{-3}$	1.72$\times$ $10^{-2}$	7.46$\times$ $10^{-3}$	1.56$\times$ $10^{-2}$	1.76$\times$ $10^{-2}$	2.57$\times$ $10^{-2}$	6.95$\times$ $10^{-3}$	1.53$\times$ $10^{-2}$

**Table 3:** Performance comparison using $\Delta _{p}$ metric across benchmark problems, with bold values indicating best results.

Test problem	M	D	MOHEOA		NSGA-II		MOChOA		MOPSO		MOEA/D		SPEA2	
			Mean	SD	Mean	SD	Mean	SD	Mean	SD	Mean	SD	Mean	SD
ZDT														
ZDT1	2	30	**1.17**$\times$ $10^{-3}$	**3.30**$\times$ $10^{-4}$	5.11$\times$ $10^{-1}$	1.09$\times$ $10^{-1}$	1.04$\times$ $10^{-2}$	5.67$\times$ $10^{-3}$	1.50$\times$ $10^{-3}$	2.96$\times$ $10^{-4}$	1.85$\times$ $10^{-3}$	3.67$\times$ $10^{-4}$	2.50$\times$ $10^{-3}$	1.48$\times$ $10^{-2}$
ZDT2	2	30	1.35$\times$ $10^{-3}$	4.82$\times$ $10^{-4}$	9.07$\times$ $10^{-1}$	4.09$\times$ $10^{-1}$	**4.77**$\times$ $10^{-4}$	**3.86**$\times$ $10^{-4}$	1.74$\times$ $10^{-3}$	3.70$\times$ $10^{-4}$	2.58$\times$ $10^{-3}$	8.90$\times$ $10^{-4}$	3.17$\times$ $10^{-3}$	1.20$\times$ $10^{-3}$
ZDT3	2	30	**6.97**$\times$ $10^{-4}$	**1.91**$\times$ $10^{-4}$	5.75$\times$ $10^{-2}$	1.47$\times$ $10^{-2}$	1.64$\times$ $10^{-2}$	5.00$\times$ $10^{-3}$	1.32$\times$ $10^{-3}$	1.53$\times$ $10^{-3}$	1.08$\times$ $10^{-3}$	3.00$\times$ $10^{-4}$	8.52$\times$ $10^{-4}$	1.36$\times$ $10^{-3}$
ZDT4	2	10	**1.33**$\times$ $10^{-2}$	**1.93**$\times$ $10^{-2}$	8.85$\times$ $10^{0}$	5.84$\times$ $10^{0}$	8.26$\times$ $10^{-2}$	4.90$\times$ $10^{-2}$	2.50$\times$ $10^{-2}$	2.11$\times$ $10^{-2}$	1.04$\times$ $10^{0}$	3.67$\times$ $10^{-1}$	8.64$\times$ $10^{-1}$	1.13$\times$ $10^{0}$
ZDT6	2	10	**1.39**$\times$ $10^{-3}$	**5.02**$\times$ $10^{-3}$	1.77$\times$ $10^{-2}$	1.77$\times$ $10^{-2}$	1.12$\times$ $10^{-2}$	3.99$\times$ $10^{-3}$	7.43$\times$ $10^{-3}$	5.15$\times$ $10^{-3}$	3.88$\times$ $10^{-1}$	2.13$\times$ $10^{-1}$	3.11$\times$ $10^{-1}$	3.27$\times$ $10^{-1}$
IMOP														
IMOP1	2	10	**5.53**$\times$ $10^{-6}$	**3.01**$\times$ $10^{-7}$	2.43$\times$ $10^{-5}$	1.15$\times$ $10^{-5}$	5.30$\times$ $10^{-5}$	1.34$\times$ $10^{-5}$	3.17$\times$ $10^{-5}$	3.94$\times$ $10^{-5}$	1.90$\times$ $10^{-5}$	1.01$\times$ $10^{-5}$	1.29$\times$ $10^{-1}$	1.15$\times$ $10^{-1}$
IMOP2	2	10	**5.79**$\times$ $10^{-7}$	**6.25**$\times$ $10^{-7}$	1.66$\times$ $10^{-5}$	2.49$\times$ $10^{-5}$	4.76$\times$ $10^{-6}$	9.43$\times$ $10^{-7}$	6.12$\times$ $10^{-6}$	4.70$\times$ $10^{-6}$	9.68$\times$ $10^{-6}$	1.48$\times$ $10^{-5}$	3.06$\times$ $10^{-3}$	5.63$\times$ $10^{-3}$
IMOP3	2	10	3.61$\times$ $10^{-3}$	2.64$\times$ $10^{-3}$	**9.98**$\times$ $10^{-4}$	**1.12**$\times$ $10^{-3}$	2.06$\times$ $10^{-3}$	1.31$\times$ $10^{-3}$	1.72$\times$ $10^{-3}$	1.50$\times$ $10^{-3}$	1.56$\times$ $10^{-3}$	2.32$\times$ $10^{-3}$	3.60$\times$ $10^{-1}$	2.69$\times$ $10^{-1}$
IMOP4	3	10	**3.57**$\times$ $10^{-5}$	**8.61**$\times$ $10^{-5}$	1.49$\times$ $10^{-4}$	5.78$\times$ $10^{-5}$	9.62$\times$ $10^{-5}$	8.02$\times$ $10^{-5}$	1.65$\times$ $10^{-4}$	1.00$\times$ $10^{-4}$	7.23$\times$ $10^{-5}$	4.52$\times$ $10^{-5}$	6.60$\times$ $10^{-4}$	3.51$\times$ $10^{-3}$
IMOP5	3	10	**1.98**$\times$ $10^{-4}$	**1.25**$\times$ $10^{-4}$	8.02$\times$ $10^{-4}$	1.43$\times$ $10^{-4}$	5.15$\times$ $10^{-4}$	2.22$\times$ $10^{-4}$	7.83$\times$ $10^{-4}$	1.58$\times$ $10^{-4}$	7.02$\times$ $10^{-4}$	1.33$\times$ $10^{-4}$	7.16$\times$ $10^{-3}$	2.11$\times$ $10^{-3}$
IMOP6	3	10	**4.76**$\times$ $10^{-4}$	**6.67**$\times$ $10^{-5}$	9.26$\times$ $10^{-4}$	2.18$\times$ $10^{-4}$	2.10$\times$ $10^{-3}$	2.50$\times$ $10^{-3}$	8.90$\times$ $10^{-4}$	3.82$\times$ $10^{-4}$	7.57$\times$ $10^{-4}$	1.65$\times$ $10^{-4}$	9.16$\times$ $10^{-3}$	2.38$\times$ $10^{-3}$
IMOP7	3	10	**4.96**$\times$ $10^{-6}$	**3.85**$\times$ $10^{-6}$	2.88$\times$ $10^{-4}$	7.46$\times$ $10^{-5}$	1.51$\times$ $10^{-4}$	1.09$\times$ $10^{-4}$	2.53$\times$ $10^{-4}$	3.39$\times$ $10^{-5}$	2.62$\times$ $10^{-4}$	4.55$\times$ $10^{-5}$	2.26$\times$ $10^{-1}$	5.25$\times$ $10^{-2}$
IMOP8	3	10	**5.52**$\times$ $10^{-3}$	**3.82**$\times$ $10^{-4}$	8.69$\times$ $10^{-3}$	1.62$\times$ $10^{-3}$	1.47$\times$ $10^{-2}$	3.52$\times$ $10^{-3}$	7.39$\times$ $10^{-3}$	1.98$\times$ $10^{-3}$	6.58$\times$ $10^{-3}$	1.44$\times$ $10^{-3}$	3.46$\times$ $10^{-2}$	1.52$\times$ $10^{-3}$
UF														
UF1	2	30	**5.97**$\times$ $10^{-3}$	**8.60**$\times$ $10^{-3}$	5.35$\times$ $10^{-2}$	1.60$\times$ $10^{-2}$	1.66$\times$ $10^{-2}$	1.83$\times$ $10^{-2}$	7.33$\times$ $10^{-3}$	2.30$\times$ $10^{-3}$	8.06$\times$ $10^{-3}$	1.66$\times$ $10^{-2}$	2.62$\times$ $10^{-2}$	3.70$\times$ $10^{-2}$
UF2	2	30	**2.29**$\times$ $10^{-3}$	**6.41**$\times$ $10^{-3}$	4.14$\times$ $10^{-3}$	1.10$\times$ $10^{-3}$	5.68$\times$ $10^{-3}$	3.06$\times$ $10^{-3}$	3.71$\times$ $10^{-3}$	9.04$\times$ $10^{-4}$	4.73$\times$ $10^{-3}$	9.96$\times$ $10^{-4}$	6.40$\times$ $10^{-3}$	1.69$\times$ $10^{-3}$
UF3	2	30	**2.45**$\times$ $10^{-2}$	**9.50**$\times$ $10^{-3}$	5.82$\times$ $10^{-2}$	1.07$\times$ $10^{-2}$	2.69$\times$ $10^{-2}$	2.34$\times$ $10^{-1}$	6.04$\times$ $10^{-2}$	1.09$\times$ $10^{-2}$	7.27$\times$ $10^{-2}$	1.46$\times$ $10^{-2}$	7.25$\times$ $10^{-2}$	1.35$\times$ $10^{-2}$
UF4	2	30	**7.89**$\times$ $10^{-3}$	**4.53**$\times$ $10^{-4}$	8.04$\times$ $10^{-3}$	4.09$\times$ $10^{-4}$	3.64$\times$ $10^{-1}$	2.24$\times$ $10^{-3}$	1.92$\times$ $10^{-2}$	1.45$\times$ $10^{-3}$	8.29$\times$ $10^{-3}$	4.17$\times$ $10^{-4}$	1.09$\times$ $10^{-2}$	1.27$\times$ $10^{-3}$
UF5	2	30	4.85$\times$ $10^{-1}$	2.16$\times$ $10^{-1}$	1.98$\times$ $10^{-1}$	6.90$\times$ $10^{-2}$	3.18$\times$ $10^{0}$	8.87$\times$ $10^{-2}$	1.96$\times$ $10^{-1}$	8.23$\times$ $10^{-2}$	**1.42**$\times$ $10^{-1}$	**5.98**$\times$ $10^{-2}$	1.86$\times$ $10^{-1}$	6.78$\times$ $10^{-2}$
UF6	2	30	**6.70**$\times$ $10^{-2}$	**3.65**$\times$ $10^{-2}$	8.79$\times$ $10^{-1}$	3.31$\times$ $10^{-1}$	3.13$\times$ $10^{-1}$	9.28$\times$ $10^{-1}$	1.04$\times$ $10^{-1}$	5.75$\times$ $10^{-2}$	1.08$\times$ $10^{-1}$	4.32$\times$ $10^{-2}$	1.18$\times$ $10^{-1}$	5.25$\times$ $10^{-2}$
UF7	2	30	**1.55**$\times$ $10^{-3}$	**5.18**$\times$ $10^{-3}$	4.26$\times$ $10^{-3}$	6.11$\times$ $10^{-3}$	4.22$\times$ $10^{-1}$	2.39$\times$ $10^{-1}$	8.62$\times$ $10^{-3}$	1.91$\times$ $10^{-2}$	1.26$\times$ $10^{-2}$	1.85$\times$ $10^{-2}$	4.36$\times$ $10^{-2}$	2.12$\times$ $10^{-2}$
UF8	3	30	**9.24**$\times$ $10^{-3}$	**8.61**$\times$ $10^{-3}$	3.54$\times$ $10^{-2}$	2.77$\times$ $10^{-2}$	2.33$\times$ $10^{-2}$	7.16$\times$ $10^{-3}$	3.29$\times$ $10^{-2}$	2.04$\times$ $10^{-2}$	3.38$\times$ $10^{-2}$	4.72$\times$ $10^{-2}$	6.81$\times$ $10^{-2}$	2.07$\times$ $10^{-2}$
UF9	3	30	**1.18**$\times$ $10^{-2}$	**3.37**$\times$ $10^{-3}$	6.65$\times$ $10^{-2}$	3.19$\times$ $10^{-2}$	3.86$\times$ $10^{-1}$	1.05$\times$ $10^{-1}$	4.61$\times$ $10^{-1}$	2.61$\times$ $10^{-1}$	3.06$\times$ $10^{-1}$	3.19$\times$ $10^{-1}$	8.68$\times$ $10^{-1}$	2.78$\times$ $10^{-1}$
UF10	3	30	**2.04**$\times$ $10^{-2}$	**1.81**$\times$ $10^{-2}$	1.63$\times$ $10^{-1}$	9.80$\times$ $10^{-2}$	5.26$\times$ $10^{-1}$	5.03$\times$ $10^{-1}$	1.50$\times$ $10^{-1}$	8.16$\times$ $10^{-2}$	9.63$\times$ $10^{-2}$	5.62$\times$ $10^{-2}$	1.82$\times$ $10^{-1}$	8.61$\times$ $10^{-2}$

**Table 4:** Performance comparison using Spread metric across benchmark problems, with bold values indicating best results.

Test problem	M	D	MOHEOA		NSGA-II		MOChOA		MOPSO		MOEA/D		SPEA2	
			Mean	SD	Mean	SD	Mean	SD	Mean	SD	Mean	SD	Mean	SD
ZDT														
ZDT1	2	30	**2.15**$\times$ $10^{-1}$	3.29$\times$ $10^{-2}$	8.23$\times$ $10^{-1}$	3.80$\times$ $10^{-2}$	3.59$\times$ $10^{-1}$	4.14$\times$ $10^{-2}$	1.11$\times$ $10^{0}$	8.91$\times$ $10^{-2}$	5.02$\times$ $10^{-1}$	4.00$\times$ $10^{-2}$	9.50$\times$ $10^{-1}$	1.77$\times$ $10^{-2}$
ZDT2	2	30	**3.40**$\times$ $10^{-1}$	1.16$\times$ $10^{-1}$	9.24$\times$ $10^{-1}$	1.17$\times$ $10^{-2}$	4.56$\times$ $10^{-1}$	1.82$\times$ $10^{-1}$	1.01$\times$ $10^{0}$	1.29$\times$ $10^{-2}$	4.91$\times$ $10^{-1}$	1.59$\times$ $10^{-1}$	9.33$\times$ $10^{-1}$	2.25$\times$ $10^{-2}$
ZDT3	2	30	**2.75**$\times$ $10^{-1}$	4.54$\times$ $10^{-2}$	8.24$\times$ $10^{-1}$	4.22$\times$ $10^{-2}$	4.04$\times$ $10^{-1}$	4.74$\times$ $10^{-2}$	1.15$\times$ $10^{0}$	1.11$\times$ $10^{-1}$	4.46$\times$ $10^{-1}$	5.93$\times$ $10^{-2}$	8.85$\times$ $10^{-1}$	3.62$\times$ $10^{-2}$
ZDT4	2	10	**8.58**$\times$ $10^{-1}$	1.43$\times$ $10^{-1}$	1.01$\times$ $10^{0}$	1.35$\times$ $10^{-2}$	8.96$\times$ $10^{-1}$	1.66$\times$ $10^{-1}$	1.13$\times$ $10^{0}$	8.21$\times$ $10^{-2}$	8.91$\times$ $10^{-1}$	9.41$\times$ $10^{-2}$	1.42$\times$ $10^{0}$	4.66$\times$ $10^{-1}$
ZDT6	2	10	**6.94**$\times$ $10^{-1}$	1.38$\times$ $10^{-1}$	1.20$\times$ $10^{0}$	2.03$\times$ $10^{-1}$	7.94$\times$ $10^{-1}$	1.38$\times$ $10^{-1}$	1.01$\times$ $10^{0}$	2.07$\times$ $10^{-1}$	8.32$\times$ $10^{-1}$	1.60$\times$ $10^{-1}$	1.04$\times$ $10^{0}$	2.80$\times$ $10^{-1}$
IMOP														
IMOP1	2	10	**6.91**$\times$ $10^{-1}$	1.85$\times$ $10^{-1}$	1.04$\times$ $10^{0}$	9.17$\times$ $10^{-2}$	7.23$\times$ $10^{-1}$	1.46$\times$ $10^{-1}$	9.70$\times$ $10^{-1}$	2.14$\times$ $10^{-2}$	1.12$\times$ $10^{0}$	1.31$\times$ $10^{-1}$	1.06$\times$ $10^{0}$	9.81$\times$ $10^{-2}$
IMOP2	2	10	**8.41**$\times$ $10^{-1}$	3.79$\times$ $10^{-2}$	1.01$\times$ $10^{0}$	8.96$\times$ $10^{-2}$	9.26$\times$ $10^{-1}$	5.29$\times$ $10^{-2}$	1.00$\times$ $10^{0}$	1.26$\times$ $10^{-6}$	8.90$\times$ $10^{-1}$	4.43$\times$ $10^{-2}$	9.36$\times$ $10^{-1}$	4.72$\times$ $10^{-2}$
IMOP3	2	10	**1.05**$\times$ $10^{-1}$	2.20$\times$ $10^{-1}$	1.06$\times$ $10^{0}$	1.08$\times$ $10^{-1}$	1.12$\times$ $10^{0}$	2.30$\times$ $10^{-1}$	9.68$\times$ $10^{-1}$	2.97$\times$ $10^{-2}$	**9.41**$\times$ $10^{-1}$	4.48$\times$ $10^{-2}$	1.00$\times$ $10^{0}$	4.56$\times$ $10^{-4}$
IMOP4	3	10	**7.03**$\times$ $10^{-1}$	2.05$\times$ $10^{-1}$	1.07$\times$ $10^{0}$	1.41$\times$ $10^{-1}$	7.72$\times$ $10^{-1}$	1.38$\times$ $10^{-1}$	1.01$\times$ $10^{0}$	1.65$\times$ $10^{-2}$	8.58$\times$ $10^{-1}$	8.48$\times$ $10^{-2}$	9.22$\times$ $10^{-1}$	1.03$\times$ $10^{-1}$
IMOP5	3	10	**1.29**$\times$ $10^{-1}$	3.47$\times$ $10^{-2}$	9.04$\times$ $10^{-1}$	1.16$\times$ $10^{-1}$	5.05$\times$ $10^{-1}$	4.70$\times$ $10^{-2}$	1.06$\times$ $10^{0}$	6.08$\times$ $10^{-2}$	2.72$\times$ $10^{1}$	4.21$\times$ $10^{-2}$	7.73$\times$ $10^{-1}$	7.41$\times$ $10^{-2}$
IMOP6	3	10	**2.77**$\times$ $10^{-1}$	2.03$\times$ $10^{-1}$	8.31$\times$ $10^{-1}$	9.56$\times$ $10^{-2}$	6.46$\times$ $10^{-1}$	7.73$\times$ $10^{-2}$	9.75$\times$ $10^{-1}$	1.30$\times$ $10^{-1}$	3.90$\times$ $10^{-1}$	1.66$\times$ $10^{-1}$	9.00$\times$ $10^{-1}$	1.24$\times$ $10^{-1}$
IMOP7	3	10	**9.71**$\times$ $10^{-1}$	2.87$\times$ $10^{-2}$	9.96$\times$ $10^{-1}$	1.25$\times$ $10^{-2}$	9.77$\times$ $10^{-1}$	2.63$\times$ $10^{-2}$	1.00$\times$ $10^{0}$	2.19$\times$ $10^{-5}$	9.73$\times$ $10^{-1}$	4.44$\times$ $10^{-2}$	1.01$\times$ $10^{0}$	1.61$\times$ $10^{-2}$
IMOP8	3	10	**1.54**$\times$ $10^{-1}$	1.21$\times$ $10^{-1}$	4.52$\times$ $10^{-1}$	7.73$\times$ $10^{-2}$	4.84$\times$ $10^{-1}$	9.43$\times$ $10^{-2}$	1.09$\times$ $10^{0}$	1.85$\times$ $10^{-2}$	2.81$\times$ $10^{-1}$	5.04$\times$ $10^{-2}$	6.52$\times$ $10^{-1}$	3.14$\times$ $10^{-2}$
UF														
UF1	2	30	**1.02**$\times$ $10^{-2}$	1.01$\times$ $10^{-1}$	8.51$\times$ $10^{-1}$	6.53$\times$ $10^{-2}$	1.01$\times$ $10^{0}$	1.24$\times$ $10^{-1}$	1.01$\times$ $10^{0}$	4.70$\times$ $10^{-2}$	1.00$\times$ $10^{0}$	6.43$\times$ $10^{-2}$	1.01$\times$ $10^{0}$	4.49$\times$ $10^{-2}$
UF2	2	30	**4.81**$\times$ $10^{-1}$	6.58$\times$ $10^{-2}$	7.34$\times$ $10^{-1}$	5.04$\times$ $10^{-2}$	5.42$\times$ $10^{-1}$	6.36$\times$ $10^{-2}$	9.28$\times$ $10^{-1}$	6.84$\times$ $10^{-2}$	7.10$\times$ $10^{-1}$	8.44$\times$ $10^{-2}$	9.92$\times$ $10^{-1}$	6.23$\times$ $10^{-3}$
UF3	2	30	**9.55**$\times$ $10^{-1}$	4.11$\times$ $10^{-2}$	**9.57**$\times$ $10^{-1}$	3.60$\times$ $10^{-2}$	9.64$\times$ $10^{-1}$	4.07$\times$ $10^{-2}$	1.01$\times$ $10^{0}$	1.36$\times$ $10^{-2}$	9.67$\times$ $10^{-1}$	4.81$\times$ $10^{-2}$	9.97$\times$ $10^{-1}$	4.74$\times$ $10^{-2}$
UF4	2	30	5.78$\times$ $10^{-1}$	7.44$\times$ $10^{-2}$	6.53$\times$ $10^{-1}$	3.84$\times$ $10^{-2}$	**4.89**$\times$ $10^{-1}$	4.58$\times$ $10^{-2}$	5.48$\times$ $10^{-1}$	8.08$\times$ $10^{-2}$	4.93$\times$ $10^{-1}$	1.02$\times$ $10^{-1}$	9.81$\times$ $10^{-1}$	2.28$\times$ $10^{-2}$
UF5	2	30	**9.91**$\times$ $10^{-1}$	5.89$\times$ $10^{-2}$	**9.93**$\times$ $10^{-1}$	5.91$\times$ $10^{-2}$	1.04$\times$ $10^{0}$	7.86$\times$ $10^{-2}$	1.01$\times$ $10^{0}$	1.25$\times$ $10^{-2}$	1.04$\times$ $10^{0}$	7.58$\times$ $10^{-2}$	1.05$\times$ $10^{0}$	5.67$\times$ $10^{-2}$
UF6	2	30	**9.55**$\times$ $10^{-1}$	4.70$\times$ $10^{-2}$	**9.60**$\times$ $10^{-1}$	5.62$\times$ $10^{-2}$	9.95$\times$ $10^{-1}$	8.69$\times$ $10^{-2}$	1.01$\times$ $10^{0}$	1.98$\times$ $10^{-2}$	9.78$\times$ $10^{-1}$	7.46$\times$ $10^{-2}$	1.03$\times$ $10^{0}$	8.52$\times$ $10^{-2}$
UF7	2	30	**7.93**$\times$ $10^{-1}$	2.29$\times$ $10^{-1}$	8.67$\times$ $10^{-1}$	1.27$\times$ $10^{-1}$	8.31$\times$ $10^{-1}$	2.84$\times$ $10^{-1}$	9.94$\times$ $10^{-1}$	2.90$\times$ $10^{-2}$	8.77$\times$ $10^{-1}$	1.70$\times$ $10^{-1}$	1.06$\times$ $10^{0}$	1.10$\times$ $10^{-1}$
UF8	3	30	**2.74**$\times$ $10^{-1}$	5.13$\times$ $10^{-2}$	5.25$\times$ $10^{-1}$	6.61$\times$ $10^{-2}$	6.15$\times$ $10^{-1}$	5.71$\times$ $10^{-2}$	9.05$\times$ $10^{-1}$	1.41$\times$ $10^{-1}$	4.75$\times$ $10^{-1}$	1.55$\times$ $10^{-1}$	7.38$\times$ $10^{-1}$	8.38$\times$ $10^{-2}$
UF9	3	30	**4.17**$\times$ $10^{-1}$	7.32$\times$ $10^{-2}$	7.03$\times$ $10^{-1}$	7.51$\times$ $10^{-2}$	6.44$\times$ $10^{-1}$	5.91$\times$ $10^{-2}$	8.99$\times$ $10^{-1}$	5.38$\times$ $10^{-2}$	7.05$\times$ $10^{-1}$	9.94$\times$ $10^{-2}$	9.06$\times$ $10^{-1}$	1.19$\times$ $10^{-1}$
UF10	3	30	**6.41**$\times$ $10^{-1}$	1.57$\times$ $10^{-1}$	9.34$\times$ $10^{-1}$	9.99$\times$ $10^{-2}$	7.28$\times$ $10^{-1}$	1.27$\times$ $10^{-1}$	1.02$\times$ $10^{0}$	2.87$\times$ $10^{-2}$	7.66$\times$ $10^{-1}$	8.18$\times$ $10^{-2}$	7.65$\times$ $10^{-1}$	1.16$\times$ $10^{-1}$

The $\epsilon$-indicator results presented in Table 5 further demonstrate the convergence effectiveness of the proposed MOHEOA, where smaller $\epsilon$ values indicate a closer approximation to the true Pareto front. As observed, MOHEOA achieves the lowest mean $\epsilon$ values across most benchmark problems, outperforming NSGA-II, MOChOA, MOPSO, MOEA/D, and SPEA2. In the ZDT test suite, MOHEOA consistently delivers superior convergence performance across all test cases. Similarly, for the IMOP benchmark problems, MOHEOA attains the best $\epsilon$-indicator values in the majority of cases, confirming its effectiveness in handling complex multi- and tri-objective optimization scenarios. For the UF problems, MOHEOA maintains its dominance by achieving the lowest $\epsilon$ values across nearly all test instances. Additionally, the lower or comparable standard deviation values indicate stable and reliable convergence behavior across independent runs. Furthermore, the results with respect to the spacing metric are presented in Table 6. The spacing metric measures the distribution uniformity of solutions on the Pareto front, where a smaller spacing value indicates better distribution characteristics. It is evident that MOHEOA achieves the most competitive results across the majority of test functions, producing lower spacing values than other algorithms in both bi-objective and tri-objective cases. This demonstrates that MOHEOA not only converges effectively towards the Pareto front but also distributes solutions more evenly compared to its competitors. While certain algorithms such as NSGA-II and MOPSO occasionally show comparable performance in selective test instances, the overall consistency and stability of MOHEOA in achieving low spacing values highlight its superiority in maintaining diversity and balanced solution distribution.

**Table 5:** Performance comparison using $\epsilon$-indicator across benchmark problems, with bold values indicating best results.

Test problem	M	D	MOHEOA		NSGA-II		MOChOA		MOPSO		MOEA/D		SPEA2	
			Mean	SD	Mean	SD	Mean	SD	Mean	SD	Mean	SD	Mean	SD
ZDT														
ZDT1	2	30	**1.26**$\times$ $10^{-2}$	**2.05**$\times$ $10^{-3}$	5.69$\times$ $10^{-1}$	9.94$\times$ $10^{-2}$	1.65$\times$ $10^{-1}$	8.61$\times$ $10^{-2}$	1.54$\times$ $10^{-2}$	2.80$\times$ $10^{-3}$	1.86$\times$ $10^{-2}$	3.21$\times$ $10^{-3}$	2.25$\times$ $10^{-1}$	1.00$\times$ $10^{-2}$
ZDT2	2	30	**2.58**$\times$ $10^{-2}$	**2.28**$\times$ $10^{-2}$	1.38$\times$ $10^{0}$	3.38$\times$ $10^{-1}$	5.26$\times$ $10^{-1}$	9.03$\times$ $10^{-2}$	4.87$\times$ $10^{-2}$	8.66$\times$ $10^{-2}$	4.21$\times$ $10^{-2}$	5.80$\times$ $10^{-2}$	2.20$\times$ $10^{-1}$	4.10$\times$ $10^{-3}$
ZDT3	2	30	**1.10**$\times$ $10^{-2}$	**5.72**$\times$ $10^{-3}$	4.47$\times$ $10^{-1}$	1.39$\times$ $10^{-1}$	1.62$\times$ $10^{-1}$	4.96$\times$ $10^{-2}$	1.57$\times$ $10^{-2}$	7.83$\times$ $10^{-3}$	1.81$\times$ $10^{-2}$	9.30$\times$ $10^{-3}$	2.40$\times$ $10^{-1}$	2.51$\times$ $10^{-2}$
ZDT4	2	10	**2.09**$\times$ $10^{-1}$	**1.29**$\times$ $10^{-1}$	1.09$\times$ $10^{1}$	6.19$\times$ $10^{0}$	5.24$\times$ $10^{-1}$	2.02$\times$ $10^{-1}$	2.41$\times$ $10^{-1}$	1.38$\times$ $10^{-1}$	5.19$\times$ $10^{-1}$	3.67$\times$ $10^{-1}$	6.07$\times$ $10^{-1}$	2.54$\times$ $10^{-1}$
ZDT6	2	10	**5.16**$\times$ $10^{-2}$	**2.40**$\times$ $10^{-2}$	2.52$\times$ $10^{-1}$	2.36$\times$ $10^{-1}$	8.19$\times$ $10^{-2}$	2.97$\times$ $10^{-2}$	5.24$\times$ $10^{-2}$	2.97$\times$ $10^{-2}$	1.68$\times$ $10^{-1}$	5.63$\times$ $10^{-2}$	1.60$\times$ $10^{-1}$	1.81$\times$ $10^{-2}$
IMOP														
IMOP1	2	10	**3.87**$\times$ $10^{-2}$	**3.65**$\times$ $10^{-2}$	4.52$\times$ $10^{-1}$	2.91$\times$ $10^{-1}$	3.64$\times$ $10^{-1}$	6.40$\times$ $10^{-3}$	6.68$\times$ $10^{-2}$	6.01$\times$ $10^{-2}$	2.26$\times$ $10^{-1}$	4.79$\times$ $10^{-2}$	5.32$\times$ $10^{-1}$	1.49$\times$ $10^{-1}$
IMOP2	2	10	4.37$\times$ $10^{-1}$	1.32$\times$ $10^{-1}$	5.66$\times$ $10^{-1}$	1.42$\times$ $10^{-1}$	7.85$\times$ $10^{-1}$	3.68$\times$ $10^{-6}$	5.10$\times$ $10^{-1}$	5.88$\times$ $10^{-2}$	4.93$\times$ $10^{-1}$	9.33$\times$ $10^{-2}$	**3.32**$\times$ $10^{-1}$	**6.91**$\times$ $10^{-2}$
IMOP3	2	10	**2.08**$\times$ $10^{-1}$	**2.45**$\times$ $10^{-1}$	2.72$\times$ $10^{-1}$	1.62$\times$ $10^{-1}$	5.53$\times$ $10^{-1}$	8.06$\times$ $10^{-2}$	2.93$\times$ $10^{-1}$	1.73$\times$ $10^{-1}$	5.29$\times$ $10^{-1}$	2.54$\times$ $10^{-2}$	9.86$\times$ $10^{-1}$	2.69$\times$ $10^{-4}$
IMOP4	3	10	**2.50**$\times$ $10^{-1}$	**2.11**$\times$ $10^{-1}$	4.48$\times$ $10^{-1}$	3.05$\times$ $10^{-1}$	6.65$\times$ $10^{-1}$	1.03$\times$ $10^{-1}$	2.63$\times$ $10^{-1}$	1.96$\times$ $10^{-1}$	5.66$\times$ $10^{-1}$	1.71$\times$ $10^{-1}$	3.99$\times$ $10^{-1}$	1.84$\times$ $10^{-1}$
IMOP5	3	10	6.23$\times$ $10^{-2}$	1.56$\times$ $10^{-2}$	6.37$\times$ $10^{-1}$	1.17$\times$ $10^{-1}$	5.55$\times$ $10^{-1}$	9.77$\times$ $10^{-2}$	**5.06**$\times$ $10^{-2}$	**2.27**$\times$ $10^{-2}$	9.17$\times$ $10^{-2}$	3.06$\times$ $10^{-2}$	4.90$\times$ $10^{-1}$	1.51$\times$ $10^{-1}$
IMOP6	3	10	**8.95**$\times$ $10^{-2}$	**7.38**$\times$ $10^{-2}$	4.63$\times$ $10^{-1}$	1.27$\times$ $10^{-1}$	2.79$\times$ $10^{-1}$	2.23$\times$ $10^{-1}$	1.43$\times$ $10^{-1}$	1.92$\times$ $10^{-1}$	1.40$\times$ $10^{-1}$	1.65$\times$ $10^{-1}$	1.08$\times$ $10^{0}$	4.73$\times$ $10^{-1}$
IMOP7	3	10	**8.79**$\times$ $10^{-1}$	**3.68**$\times$ $10^{-2}$	9.25$\times$ $10^{-1}$	1.69$\times$ $10^{-2}$	9.39$\times$ $10^{-1}$	4.77$\times$ $10^{-5}$	8.92$\times$ $10^{-1}$	1.18$\times$ $10^{-2}$	9.00$\times$ $10^{-1}$	2.31$\times$ $10^{-2}$	1.13$\times$ $10^{0}$	3.44$\times$ $10^{-2}$
IMOP8	3	10	**1.03**$\times$ $10^{-1}$	**2.32**$\times$ $10^{-2}$	1.94$\times$ $10^{-1}$	6.01$\times$ $10^{-2}$	1.06$\times$ $10^{0}$	5.01$\times$ $10^{-3}$	1.20$\times$ $10^{-1}$	1.13$\times$ $10^{-1}$	1.32$\times$ $10^{-1}$	7.65$\times$ $10^{-2}$	4.29$\times$ $10^{-1}$	1.62$\times$ $10^{-3}$
UF														
UF1	2	30	**1.23**$\times$ $10^{-1}$	**2.62**$\times$ $10^{-2}$	2.99$\times$ $10^{-1}$	5.10$\times$ $10^{-2}$	3.02$\times$ $10^{-1}$	1.02$\times$ $10^{-1}$	1.29$\times$ $10^{-1}$	3.45$\times$ $10^{-2}$	1.26$\times$ $10^{-1}$	3.51$\times$ $10^{-2}$	3.40$\times$ $10^{-1}$	2.40$\times$ $10^{-2}$
UF2	2	30	6.36$\times$ $10^{-2}$	1.17$\times$ $10^{-2}$	1.08$\times$ $10^{-1}$	2.33$\times$ $10^{-2}$	2.17$\times$ $10^{-1}$	5.77$\times$ $10^{-2}$	6.47$\times$ $10^{-2}$	7.75$\times$ $10^{-3}$	6.85$\times$ $10^{-2}$	7.52$\times$ $10^{-3}$	2.93$\times$ $10^{-1}$	1.62$\times$ $10^{-2}$
UF3	2	30	3.86$\times$ $10^{-1}$	5.31$\times$ $10^{-2}$	3.29$\times$ $10^{-1}$	4.10$\times$ $10^{-2}$	3.27$\times$ $10^{-1}$	2.25$\times$ $10^{-2}$	3.91$\times$ $10^{-1}$	5.19$\times$ $10^{-2}$	3.98$\times$ $10^{-1}$	4.81$\times$ $10^{-2}$	3.99$\times$ $10^{-1}$	2.99$\times$ $10^{-2}$
UF4	2	30	**7.30**$\times$ $10^{-2}$	**3.38**$\times$ $10^{-3}$	1.59$\times$ $10^{-1}$	1.41$\times$ $10^{-2}$	1.27$\times$ $10^{-1}$	6.46$\times$ $10^{-3}$	7.46$\times$ $10^{-2}$	3.44$\times$ $10^{-3}$	7.70$\times$ $10^{-2}$	2.70$\times$ $10^{-3}$	2.19$\times$ $10^{-1}$	1.76$\times$ $10^{-3}$
UF5	2	30	7.23$\times$ $10^{-1}$	1.50$\times$ $10^{-1}$	1.77$\times$ $10^{0}$	5.03$\times$ $10^{-1}$	1.38$\times$ $10^{0}$	2.97$\times$ $10^{-1}$	7.55$\times$ $10^{-1}$	2.28$\times$ $10^{-1}$	5.73$\times$ $10^{-1}$	1.48$\times$ $10^{-1}$	7.15$\times$ $10^{-1}$	2.24$\times$ $10^{-1}$
UF6	2	30	**3.83**$\times$ $10^{-1}$	**1.17**$\times$ $10^{-1}$	9.34$\times$ $10^{-1}$	3.00$\times$ $10^{-1}$	5.41$\times$ $10^{-1}$	1.57$\times$ $10^{-1}$	4.21$\times$ $10^{-1}$	1.29$\times$ $10^{-1}$	4.52$\times$ $10^{-1}$	9.53$\times$ $10^{-2}$	4.87$\times$ $10^{-1}$	8.75$\times$ $10^{-2}$
UF7	2	30	**1.91**$\times$ $10^{-1}$	**1.36**$\times$ $10^{-1}$	3.47$\times$ $10^{-1}$	1.15$\times$ $10^{-1}$	4.99$\times$ $10^{-1}$	1.36$\times$ $10^{-1}$	2.04$\times$ $10^{-1}$	1.28$\times$ $10^{-1}$	2.02$\times$ $10^{-1}$	1.28$\times$ $10^{-1}$	3.26$\times$ $10^{-1}$	6.05$\times$ $10^{-2}$
UF8	3	30	**2.65**$\times$ $10^{-1}$	**1.17**$\times$ $10^{-2}$	3.29$\times$ $10^{-1}$	6.47$\times$ $10^{-2}$	6.28$\times$ $10^{-1}$	2.51$\times$ $10^{-1}$	3.04$\times$ $10^{-1}$	2.54$\times$ $10^{-2}$	3.21$\times$ $10^{-1}$	5.42$\times$ $10^{-2}$	5.11$\times$ $10^{-1}$	2.59$\times$ $10^{-2}$
UF9	3	30	**4.07**$\times$ $10^{-1}$	**5.85**$\times$ $10^{-2}$	5.25$\times$ $10^{-1}$	5.20$\times$ $10^{-2}$	5.17$\times$ $10^{-1}$	8.89$\times$ $10^{-2}$	4.20$\times$ $10^{-1}$	6.02$\times$ $10^{-2}$	4.16$\times$ $10^{-1}$	6.93$\times$ $10^{-2}$	5.61$\times$ $10^{-1}$	4.99$\times$ $10^{-2}$
UF10	3	30	**6.75**$\times$ $10^{-1}$	**2.27**$\times$ $10^{-1}$	8.05$\times$ $10^{-1}$	1.22$\times$ $10^{-1}$	7.39$\times$ $10^{-1}$	8.20$\times$ $10^{-2}$	9.31$\times$ $10^{-1}$	4.18$\times$ $10^{-1}$	1.01$\times$ $10^{0}$	5.34$\times$ $10^{-1}$	1.07$\times$ $10^{0}$	4.51$\times$ $10^{-1}$

**Table 6:** Performance comparison using Spacing indicator across benchmark problems, with bold values indicating best results.

Test problem	M	D	MOHEOA		NSGAII		MOChOA		MOPSO		MOEA/D		SPEA2	
			Mean	SD	Mean	SD	Mean	SD	Mean	SD	Mean	SD	Mean	SD
ZDT														
ZDT1	2	30	**4.66**$\times$ $10^{-3}$	**6.14**$\times$ $10^{-4}$	1.22$\times$ $10^{-2}$	1.22$\times$ $10^{-3}$	7.32$\times$ $10^{-3}$	7.30$\times$ $10^{-4}$	9.07$\times$ $10^{-3}$	1.09$\times$ $10^{-3}$	1.56$\times$ $10^{-2}$	1.04$\times$ $10^{-2}$	6.63$\times$ $10^{-3}$	6.17$\times$ $10^{-4}$
ZDT2	2	30	**1.19**$\times$ $10^{-3}$	**1.87**$\times$ $10^{-3}$	9.29$\times$ $10^{-3}$	6.81$\times$ $10^{-4}$	8.58$\times$ $10^{-3}$	2.45$\times$ $10^{-3}$	1.19$\times$ $10^{-2}$	6.04$\times$ $10^{-3}$	7.55$\times$ $10^{-3}$	2.19$\times$ $10^{-3}$	6.65$\times$ $10^{-3}$	1.39$\times$ $10^{-3}$
ZDT3	2	30	**5.86**$\times$ $10^{-3}$	**8.85**$\times$ $10^{-4}$	1.30$\times$ $10^{-2}$	2.49$\times$ $10^{-3}$	7.97$\times$ $10^{-3}$	7.98$\times$ $10^{-4}$	9.91$\times$ $10^{-3}$	1.04$\times$ $10^{-3}$	2.89$\times$ $10^{-2}$	1.71$\times$ $10^{-2}$	7.22$\times$ $10^{-3}$	6.66$\times$ $10^{-4}$
ZDT4	2	10	**1.88**$\times$ $10^{-2}$	**1.35**$\times$ $10^{-2}$	4.34$\times$ $10^{-1}$	8.68$\times$ $10^{-1}$	1.36$\times$ $10^{-1}$	2.01$\times$ $10^{-1}$	4.80$\times$ $10^{-2}$	3.07$\times$ $10^{-2}$	2.43$\times$ $10^{-2}$	3.28$\times$ $10^{-2}$	2.76$\times$ $10^{-2}$	2.18$\times$ $10^{-2}$
ZDT6	2	10	1.56$\times$ $10^{-2}$	3.87$\times$ $10^{-3}$	4.52$\times$ $10^{-2}$	4.44$\times$ $10^{-2}$	3.56$\times$ $10^{-2}$	7.35$\times$ $10^{-2}$	4.14$\times$ $10^{-2}$	6.47$\times$ $10^{-2}$	1.67$\times$ $10^{-2}$	1.82$\times$ $10^{-2}$	**1.11**$\times$ $10^{-2}$	**5.19**$\times$ $10^{-3}$
IMOP														
IMOP1	2	10	**1.71**$\times$ $10^{-3}$	**6.61**$\times$ $10^{-4}$	1.93$\times$ $10^{-2}$	2.77$\times$ $10^{-2}$	3.75$\times$ $10^{-2}$	9.24$\times$ $10^{-3}$	3.65$\times$ $10^{-2}$	2.19$\times$ $10^{-2}$	3.85$\times$ $10^{-2}$	2.64$\times$ $10^{-2}$	3.48$\times$ $10^{-2}$	1.88$\times$ $10^{-2}$
IMOP2	2	10	**2.02**$\times$ $10^{-7}$	**4.41**$\times$ $10^{-7}$	6.92$\times$ $10^{-3}$	2.22$\times$ $10^{-2}$	8.55$\times$ $10^{-2}$	2.91$\times$ $10^{-2}$	1.99$\times$ $10^{-3}$	1.17$\times$ $10^{-3}$	9.81$\times$ $10^{-4}$	6.43$\times$ $10^{-4}$	7.63$\times$ $10^{-3}$	2.06$\times$ $10^{-2}$
IMOP3	2	10	**4.29**$\times$ $10^{-3}$	**1.96**$\times$ $10^{-3}$	3.26$\times$ $10^{-2}$	2.23$\times$ $10^{-2}$	5.37$\times$ $10^{-2}$	1.73$\times$ $10^{-2}$	1.63$\times$ $10^{-2}$	2.82$\times$ $10^{-2}$	4.03$\times$ $10^{-2}$	3.89$\times$ $10^{-2}$	5.11$\times$ $10^{-2}$	4.75$\times$ $10^{-2}$
IMOP4	3	10	**5.74**$\times$ $10^{-3}$	**3.14**$\times$ $10^{-3}$	5.00$\times$ $10^{-2}$	6.15$\times$ $10^{-2}$	6.37$\times$ $10^{-2}$	2.19$\times$ $10^{-2}$	3.65$\times$ $10^{-2}$	5.47$\times$ $10^{-2}$	5.15$\times$ $10^{-2}$	6.39$\times$ $10^{-2}$	3.27$\times$ $10^{-2}$	4.00$\times$ $10^{-2}$
IMOP5	3	10	**3.56**$\times$ $10^{-3}$	**5.10**$\times$ $10^{-3}$	2.61$\times$ $10^{-2}$	6.77$\times$ $10^{-3}$	4.29$\times$ $10^{-2}$	1.03$\times$ $10^{-2}$	4.15$\times$ $10^{-2}$	8.47$\times$ $10^{-3}$	2.07$\times$ $10^{-2}$	8.50$\times$ $10^{-3}$	3.83$\times$ $10^{-2}$	6.55$\times$ $10^{-3}$
IMOP6	3	10	**1.16**$\times$ $10^{-2}$	**7.41**$\times$ $10^{-3}$	5.69$\times$ $10^{-2}$	2.04$\times$ $10^{-2}$	5.26$\times$ $10^{-2}$	6.24$\times$ $10^{-3}$	4.49$\times$ $10^{-2}$	2.48$\times$ $10^{-2}$	2.49$\times$ $10^{-2}$	2.14$\times$ $10^{-2}$	5.19$\times$ $10^{-2}$	1.32$\times$ $10^{-2}$
IMOP7	3	10	**1.23**$\times$ $10^{-5}$	**1.09**$\times$ $10^{-5}$	3.20$\times$ $10^{-4}$	5.32$\times$ $10^{-4}$	1.11$\times$ $10^{-2}$	2.06$\times$ $10^{-2}$	7.56$\times$ $10^{-4}$	6.90$\times$ $10^{-4}$	4.32$\times$ $10^{-4}$	5.67$\times$ $10^{-4}$	1.50$\times$ $10^{-3}$	1.82$\times$ $10^{-3}$
IMOP8	3	10	**4.22**$\times$ $10^{-2}$	**1.02**$\times$ $10^{-2}$	7.84$\times$ $10^{-2}$	1.34$\times$ $10^{-2}$	9.50$\times$ $10^{-2}$	1.03$\times$ $10^{-2}$	9.91$\times$ $10^{-2}$	1.71$\times$ $10^{-2}$	4.25$\times$ $10^{-2}$	7.46$\times$ $10^{-3}$	8.96$\times$ $10^{-2}$	1.64$\times$ $10^{-2}$
UF														
UF1	2	30	**6.11**$\times$ $10^{-3}$	**7.63**$\times$ $10^{-3}$	1.59$\times$ $10^{-2}$	8.37$\times$ $10^{-3}$	1.30$\times$ $10^{-2}$	1.66$\times$ $10^{-2}$	1.01$\times$ $10^{-2}$	2.35$\times$ $10^{-2}$	1.23$\times$ $10^{-2}$	1.66$\times$ $10^{-2}$	9.49$\times$ $10^{-3}$	1.86$\times$ $10^{-2}$
UF2	2	30	**7.14**$\times$ $10^{-3}$	**3.23**$\times$ $10^{-3}$	9.63$\times$ $10^{-3}$	5.50$\times$ $10^{-3}$	7.29$\times$ $10^{-3}$	1.84$\times$ $10^{-3}$	1.25$\times$ $10^{-2}$	4.82$\times$ $10^{-3}$	9.97$\times$ $10^{-3}$	5.85$\times$ $10^{-3}$	8.12$\times$ $10^{-3}$	5.75$\times$ $10^{-3}$
UF3	2	30	**2.16**$\times$ $10^{-3}$	**4.50**$\times$ $10^{-3}$	3.51$\times$ $10^{-3}$	3.38$\times$ $10^{-3}$	2.85$\times$ $10^{-2}$	3.02$\times$ $10^{-2}$	1.81$\times$ $10^{-2}$	8.99$\times$ $10^{-3}$	1.73$\times$ $10^{-2}$	1.42$\times$ $10^{-2}$	1.44$\times$ $10^{-2}$	1.01$\times$ $10^{-2}$
UF4	2	30	1.13$\times$ $10^{-2}$	3.04$\times$ $10^{-3}$	**6.73**$\times$ $10^{-3}$	**8.72**$\times$ $10^{-4}$	1.16$\times$ $10^{-2}$	2.06$\times$ $10^{-3}$	9.06$\times$ $10^{-3}$	2.52$\times$ $10^{-3}$	7.41$\times$ $10^{-3}$	3.62$\times$ $10^{-3}$	6.86$\times$ $10^{-3}$	1.68$\times$ $10^{-3}$
UF5	2	30	**5.88**$\times$ $10^{-3}$	**1.13**$\times$ $10^{-2}$	5.55$\times$ $10^{-2}$	1.67$\times$ $10^{-1}$	3.01$\times$ $10^{-2}$	3.21$\times$ $10^{-2}$	5.73$\times$ $10^{-2}$	3.50$\times$ $10^{-2}$	6.76$\times$ $10^{-2}$	4.87$\times$ $10^{-2}$	5.09$\times$ $10^{-2}$	3.79$\times$ $10^{-2}$
UF6	2	30	**4.65**$\times$ $10^{-3}$	**6.40**$\times$ $10^{-3}$	1.33$\times$ $10^{-2}$	1.81$\times$ $10^{-2}$	2.29$\times$ $10^{-2}$	2.10$\times$ $10^{-2}$	3.35$\times$ $10^{-2}$	6.49$\times$ $10^{-2}$	3.62$\times$ $10^{-2}$	5.80$\times$ $10^{-2}$	2.65$\times$ $10^{-2}$	2.01$\times$ $10^{-2}$
UF7	2	30	**1.06**$\times$ $10^{-3}$	**3.34**$\times$ $10^{-3}$	2.40$\times$ $10^{-2}$	4.09$\times$ $10^{-2}$	1.43$\times$ $10^{-2}$	1.33$\times$ $10^{-2}$	1.15$\times$ $10^{-2}$	8.72$\times$ $10^{-3}$	1.22$\times$ $10^{-2}$	2.18$\times$ $10^{-2}$	9.44$\times$ $10^{-3}$	1.47$\times$ $10^{-2}$
UF8	3	30	**2.34**$\times$ $10^{-2}$	**1.29**$\times$ $10^{-2}$	3.56$\times$ $10^{-2}$	1.16$\times$ $10^{-2}$	9.64$\times$ $10^{-2}$	3.87$\times$ $10^{-2}$	7.94$\times$ $10^{-2}$	8.60$\times$ $10^{-2}$	4.37$\times$ $10^{-2}$	9.07$\times$ $10^{-3}$	7.14$\times$ $10^{-2}$	2.53$\times$ $10^{-2}$
UF9	3	30	2.92$\times$ $10^{-2}$	4.91$\times$ $10^{-3}$	**2.37**$\times$ $10^{-2}$	**1.15**$\times$ $10^{-2}$	1.72$\times$ $10^{-1}$	9.69$\times$ $10^{-2}$	4.64$\times$ $10^{-2}$	2.88$\times$ $10^{-2}$	5.56$\times$ $10^{-2}$	3.27$\times$ $10^{-2}$	7.78$\times$ $10^{-2}$	3.09$\times$ $10^{-2}$
UF10	3	30	**1.21**$\times$ $10^{-2}$	**2.88**$\times$ $10^{-2}$	2.14$\times$ $10^{-2}$	3.72$\times$ $10^{-2}$	1.51$\times$ $10^{-1}$	5.86$\times$ $10^{-2}$	1.39$\times$ $10^{-1}$	6.22$\times$ $10^{-2}$	1.41$\times$ $10^{-1}$	4.66$\times$ $10^{-2}$	1.74$\times$ $10^{-1}$	8.04$\times$ $10^{-2}$

### Pareto front analysis

**Fig. 6:**
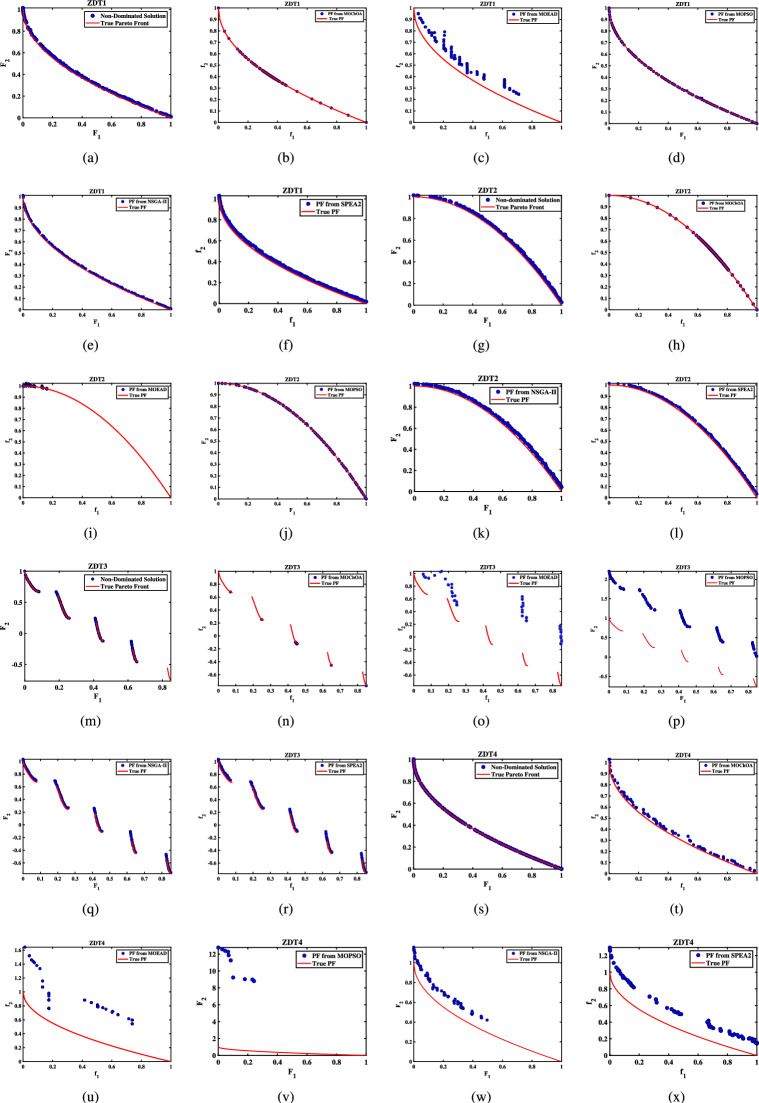
Pareto front results obtained from proposed MOHEOA and other comparative approaches for (**a**–**f**) ZDT1, (**g**–**l**) ZDT2, (**m**–**r**) ZDT3 and (**s**–**x**) ZDT4 test functions.

**Fig. 7:**
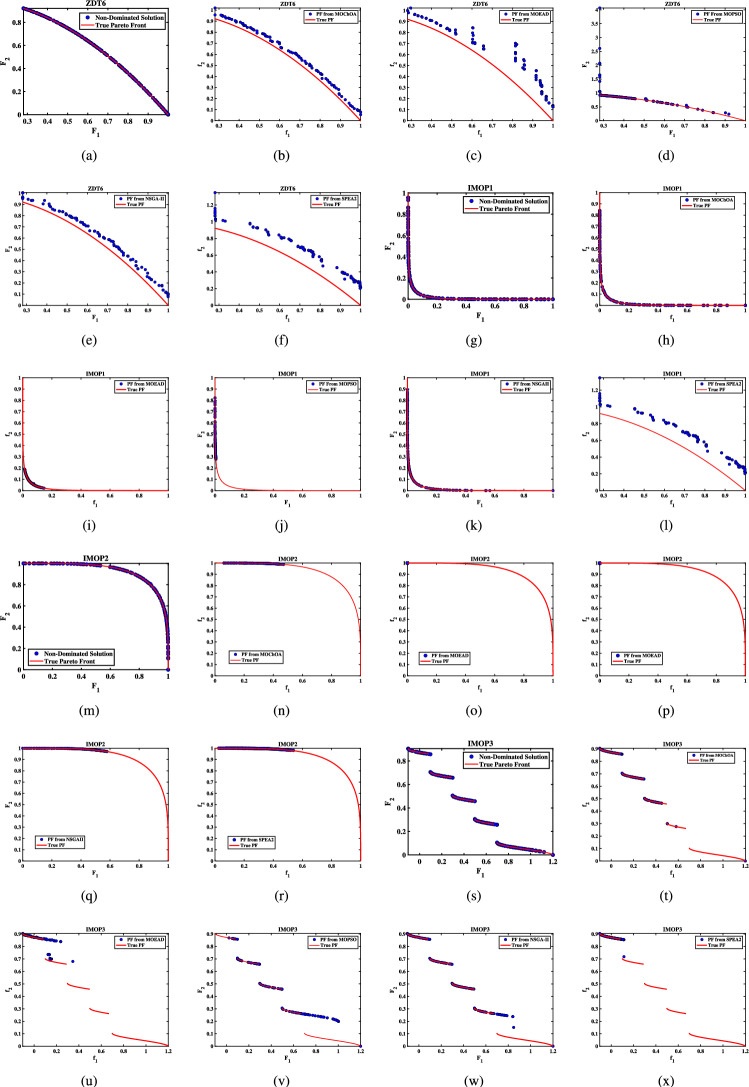
Pareto front results obtained from proposed MOHEOA and other comparative approaches for (**a**–**f**) ZDT6, (**g**–**l**) IMOP1, (**m**–**r**) IMOP2, and (**s**–**x**) IMOP3.

**Fig. 8:**
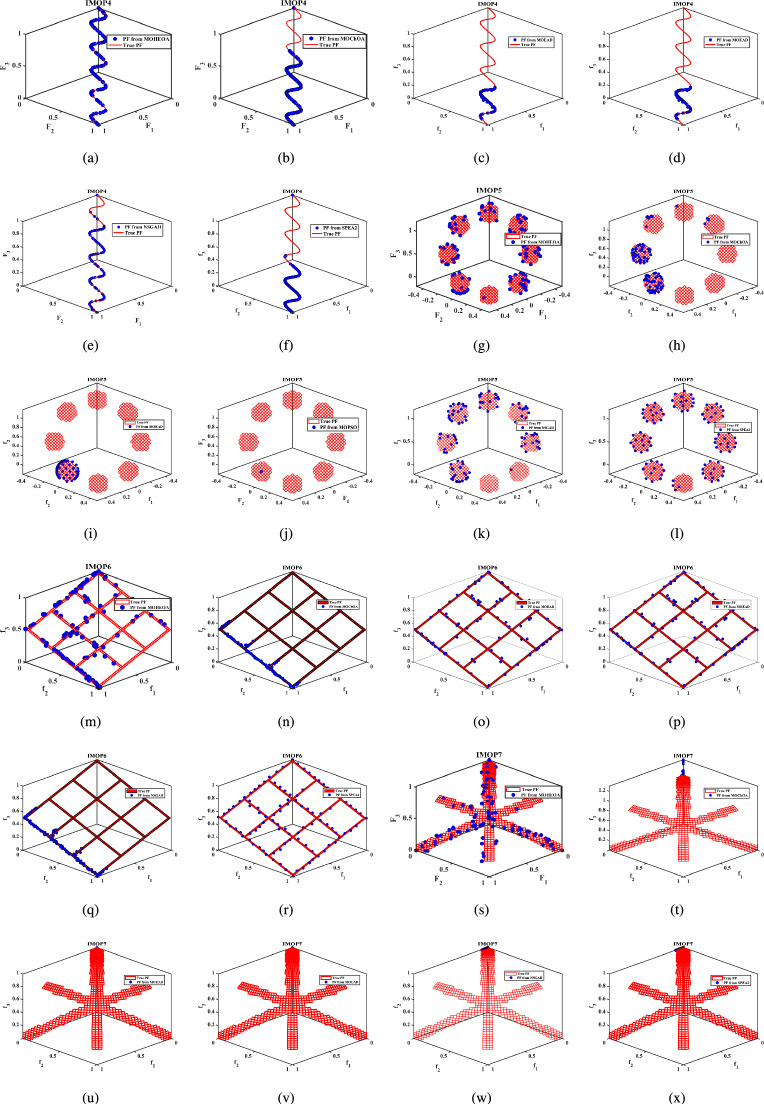
Pareto front results obtained from proposed MOHEOA and other comparative approaches for (**a**–**f**) IMOP4, (**g**–**l**) IMOP5, (**m**–**r**) IMOP6, and (**s**–**x**) IMOP7 test functions.

**Fig. 9:**
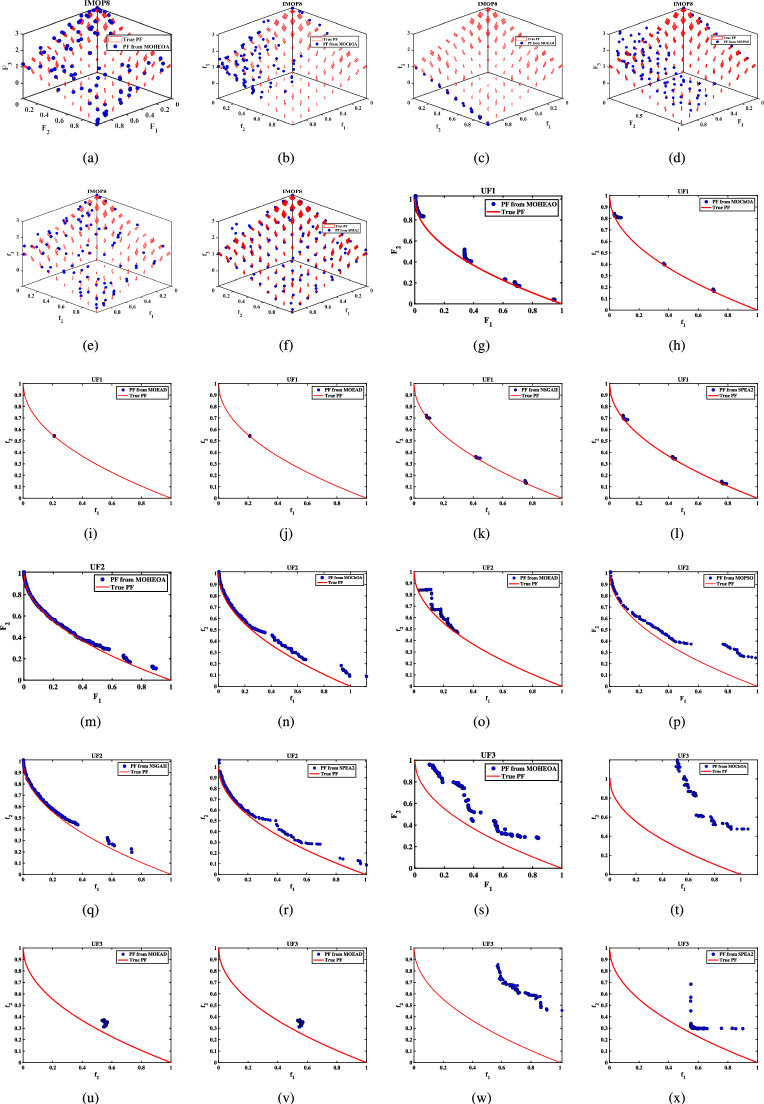
Pareto front results obtained from proposed MOHEOA and other comparative approaches for (**a**–**f**) IMOP8, (**g**–**l**) UF1, (**m**–**r**) UF2, and (**s**–**x**) UF3 test functions.

**Fig. 10:**
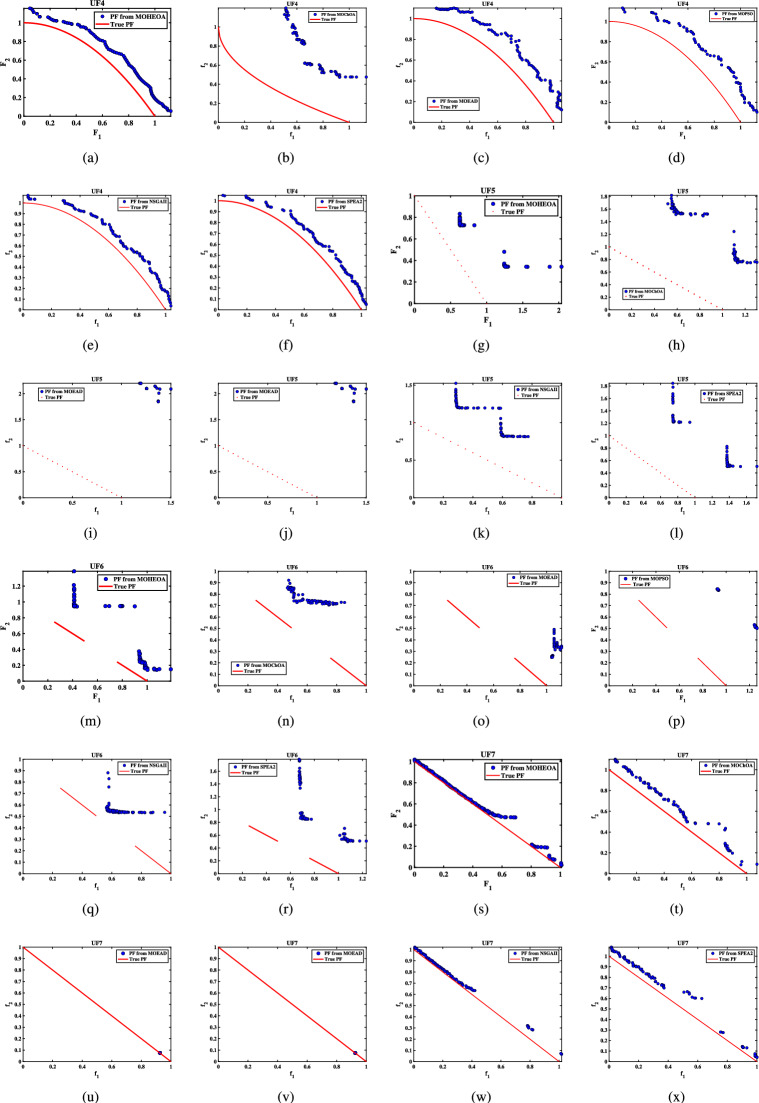
Pareto front results obtained from proposed MOHEOA and other comparative approaches for (**a**–**f**) UF4, (**g**–**l**) UF5, (**m**–**r**) UF6 and (**s**–**x**) UF7 test functions.

**Fig. 11:**
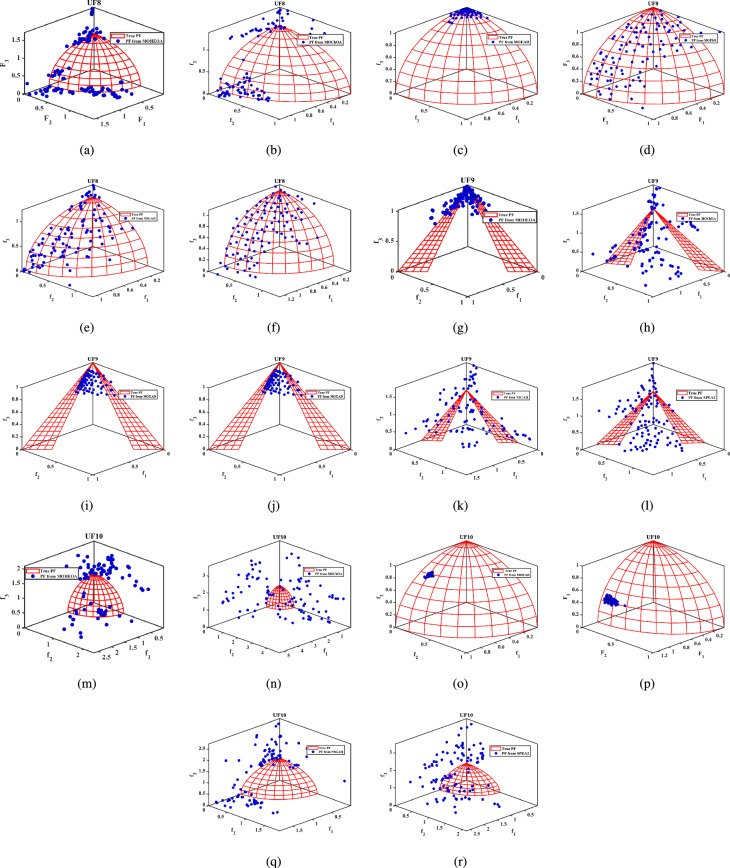
Pareto front results obtained from proposed MOHEOA and other comparative approaches for (**a**–**f**) UF8, (**g**–**l**) UF9, and (**m**–**r**) UF10 test functions.

To demonstrate optimization performance visually, the Pareto fronts obtained from 30 independent runs are plotted in Fig. 6. The figure presents Pareto front results for the ZDT1, ZDT2, ZDT3 and ZDT4 benchmark problems, comparing the proposed MOHEOA with other existing approaches. In the ZDT1 benchmark Fig. 6a–f, the True Pareto front is represented by a smooth red curve, while the non-dominated solutions obtained by different algorithms are shown as blue points. MOHEOA demonstrates superior performance by closely aligning with the true Pareto front, indicating strong convergence and diversity maintenance. In contrast, some comparative methods, such as the one in Fig. 6c, show poorly distributed solutions, highlighting their inefficiency in preserving diversity. Similarly, in the ZDT2 benchmark Fig. 6g–l, MOHEOA provides solutions that are well-spread and tightly fit the optimal Pareto front, whereas some competing algorithms struggle with convergence and diversity. The Fig. 6m—r,s–x compares MOHEOA with other methods on ZDT3 and ZDT4 benchmark problems. For ZDT3 test function, MOHEOA effectively captures the true Pareto front’s disconnected segments, while other methods struggle with diversity. In ZDT4, where the search space is highly complex, MOHEOA outperforms others by achieving well-converged, well-spread solutions, while some methods show poor distribution. The Fig. 7a–f shows Pareto front results for ZDT6, where MOHEOA achieves superior convergence and diversity, closely matching the true front. Other methods Fig. 7b–f struggle with poor distribution and deviation. The proposed MOHEOA is also tested on IMOP test functions which is one fo the most challenging problem for multi-objective optimization. Pareto front results for IMOP1 to IMOP8 depicted in Figs. 7, 8 and 9 shows the effectiveness of the proposed MOHEOA compared to other approaches. Fig. 7g–x shows the results Pareto front results obtained for IMOP1, IMOP2 and IMOP3 test functions. It is clearly visible from Fig. 7g for IMOP1, PF obtained from MOHEOA closely follows the True Pareto front with well-distributed and converged solutions, while other methods show noticeable deviations, especially struggling with maintaining diversity as depicted in Fig. 7h–l. The results for the IMOP2 test function, as shown in Fig. 7m–r, clearly highlight the superior performance of the proposed MOHEOA algorithm. Among all the tested approaches, only MOHEOA successfully converges to the true Pareto front, demonstrating its strong ability to maintain both convergence and diversity. In contrast, the other algorithms struggle significantly, failing to reach the true Pareto front and exhibiting poor solution distribution. The result for IMOP3 and IMOP4 function is depicted in Figs. 7s–x and 8a–f, respectively. Fig. 7s–x shows increasingly complex challenges with discontinuous and multimodal Pareto fronts for the IMOP3 test function, Fig. 8a clearly demonstrates that MOHEOA effectively captures the intricate structure of the true Pareto front, ensuring both convergence and diversity. In contrast, other algorithms struggle significantly, with SPEA2 and MOEA/D performing particularly poorly, failing to maintain consistent convergence and producing solutions that deviate substantially from the true Pareto front. For the IMOP5 test problem, Fig. 8g illustrates that the proposed MOHEOA achieves the closest convergence to the true Pareto front, surpassing comparative algorithms like NSGA-II, MOEA/D, MOChOA, and MOPSO. The Pareto front of IMOP5 consists of eight circles. While NSGA-II and MOChOA exhibit partial convergence, MOEA/D and MOPSO produce solutions with poor distribution. In contrast, MOHEOA maintains superior accuracy and uniformity across the entire front. IMOP6 introduces a deceptive and biased Pareto front structured as multiple grids as depicted in Fig. 8m–r. Here, MOHEOA again outperforms NSGA-II, MOEA/D, MOChOA, and MOPSO, demonstrating significantly better diversity. The competing algorithms either fail to cover the entire front or lose solution diversity, whereas MOHEOA preserves both convergence and spread. IMOP7 poses a severe challenge with its high-dimensional, deceptive, and disconnected Pareto front as shown in Fig. 8s–x. None of the compared algorithms—NSGA-II, MOEA/D, MOChOA, or MOPSO—fully cover the front, but MOHEOA achieves notably better coverage and distribution, albeit with residual gaps. Finally, IMOP8 features an even more complex Pareto front depicted in Fig. 9a–e. MOHEOA excels again, showcasing markedly better diversity and convergence than NSGA-II, MOEA/D, MOChOA, and MOPSO, which struggle with fragmented or incomplete solutions. These test problems (IMOP5–IMOP8) rank among the most demanding in the IMOP benchmark suite. MOHEOA demonstrates superior performance across UF test functions when compared to MOPSO, NSGA-II, SPEA-2, MOEA/D, MOGWO, and MOChOA as depicted in Figs. 9, 10 and 11. For UF1 and UF5, MOHEOA achieves near-perfect convergence

to the true Pareto front, outperforming MOPSO and MOEA/D, which struggle with distribution and partial coverage. On UF3 and UF4, MOHEOA maintains better solution diversity and accuracy than NSGA-II and SPEA-2, which often exhibit premature convergence. For discontinuous PFs (UF5–UF7), MOHEOA excels where others falter, MOPSO and MOChOA fail to cover fragmented regions, while MOEA/D’s decomposition approach struggles with high-dimensional discontinuities. MOGWO, though competitive, shows inferior coverage compared to MOHEOA’s balanced convergence-diversity trade-off. In three-objective problems (UF8–UF10), MOHEOA significantly outperforms MOPSO and NSGA-II in both convergence and spread, while MOEA/D is limited by its lack of a three-objective variant. SPEA-2 and MOChOA exhibit poor stability, whereas MOHEOA consistently provides well-distributed solutions near the true PF. All these results consistently highlight MOHEOA’s dominance over state-of-the-art MOEAs in handling complex Pareto fronts, particularly in terms of convergence, diversity, and robustness.

### Wilcoxon signed rank test

For a robust statistical assessment, the Wilcoxon Signed-Ranks Test (WSRT)^72^ is applied to evaluate the comparative performance of the algorithms. As a nonparametric method, WSRT determines whether two algorithms exhibit statistically significant differences in their outcomes. The null hypothesis $H_{o}$ assumes that no median difference exists between the solutions generated by Algorithm A and Algorithm B for a given test function^73^. To assess whether Algorithm A outperforms Algorithm B or if the alternative hypothesis holds, the rank sums derived from WSRT are analyzed. The test involves calculating the positive $R^{+}$ and negative $R^{-}$ rank sums before determining the corresponding *p*-values. In this study, WSRT is conducted at a significance level of $\alpha = 0.05$ to ensure rigorous multi-problem statistical evaluation. To conduct a comprehensive statistical comparison across multiple test problems, the average values of the four performance metrics—calculated over 30 independent runs for each problem—serve as the sample dataset. Tables 7 and 8 displays the results of the Wilcoxon Signed-Ranks Test (WSRT), which evaluates the statistical differences between MOHEOA and each reference algorithm. The table also presents the detailed statistical outcomes for every test problem based on the 30-run experiments. In the presented tables, the symbol “+” indicates instances where the null hypothesis is rejected, demonstrating that MOHEOA achieves statistically superior results in single-problem comparisons at a 95% confidence level $(\alpha =0.5)$. Conversely, the “-” symbol denotes cases where the null hypothesis is rejected, and also reveals that performs significantly worse. The “=” symbol signifies scenarios where no statistically detectable difference exists between MOHEOA and the competing algorithms.

**Table 7:** Comparison of MOHEOA with other algorithms using Hypervolume and $\Delta _{p}$ metrics.

	Hypervolume				$\Delta _{p}$			
Vs.	R+	R−	p-value	Sign	R+	R−	*p*-value	Sign
MOChOA	252	33	0.0026	+	255	30	0.0019	+
MOEAD	267	18	0.0004	+	231	54	0.0228	+
MOPSO	270	15	0.0003	+	203	82	0.1446	=
NSGA-II	285	0	0.0001	+	270	15	0.0003	+
SPAE2	285	0	0.0001	+	285	0	0.0001	+

**Table 8:** Comparison of MOHEOA with other algorithms using HV and Spread metrics.

	Spread				$\epsilon$			
Vs.	R+	R−	*p*-value	Sign	R+	R−	p-value	Sign
MOChOA	285	0	< 0.0001	+	163	122	0.6894	=
MOEAD	267	18	0.0004	+	145	141	0.9867	=
MOPSO	210	75	0.0833	=	229	56	0.002	+
NSGA-II	285	0	< 0.0001	+	118	167	0.2596	=
SPAE2	285	0	< 0.0001	+	228	57	0.0002	−

The Wilcoxon rank-sum test results across four key performance metrics reveal MOHEOA’s strengths and limitations compared to other multi-objective optimization algorithms. Table 7 shows the result in terms of HV metric in which MOHEOA demonstrates statistically superior performance against all competitors, with particularly dominant results against NSGA-II and SPEA2 ($R^{-}$=0, p<0.0001), indicating exceptional convergence to the true Pareto front. Similar superiority is observed for $\Delta _{p}$ metric, where MOHEOA significantly outperforms MOChOA (p=0.0019), MOEAD (p=0.0228), NSGA-II (p=0.0003), and SPEA2 (p<0.0001), while showing comparable performance to MOPSO (p=0.1446). The spread metric further confirms MOHEOA’s effectiveness, with significant advantages over MOChOA, MOEAD, NSGA-II, and SPEA2 (all p$\ge$0.0004), though no significant difference from MOPSO (p=0.0833), suggesting equally good solution coverage in some cases. However, the $\epsilon$ metric reveals a trade-off: while MOHEOA maintains comparable diversity to MOChOA, MOEAD and NSGA-II (all p$\ge$0.25), it is significantly outperformed by MOPSO (p=0.002) and SPEA2 (p=0.0002) in solution distribution uniformity. MOHEOA’s statistically significant superiority, assessed by WSRT, is maintained across both bi-objective and tri-objective problems, as detailed in the results tables and figures, with performance variations fully documented and consistently favoring MOHEOA in terms of the four metrics.

### Box plot analysis

**Fig. 12:**
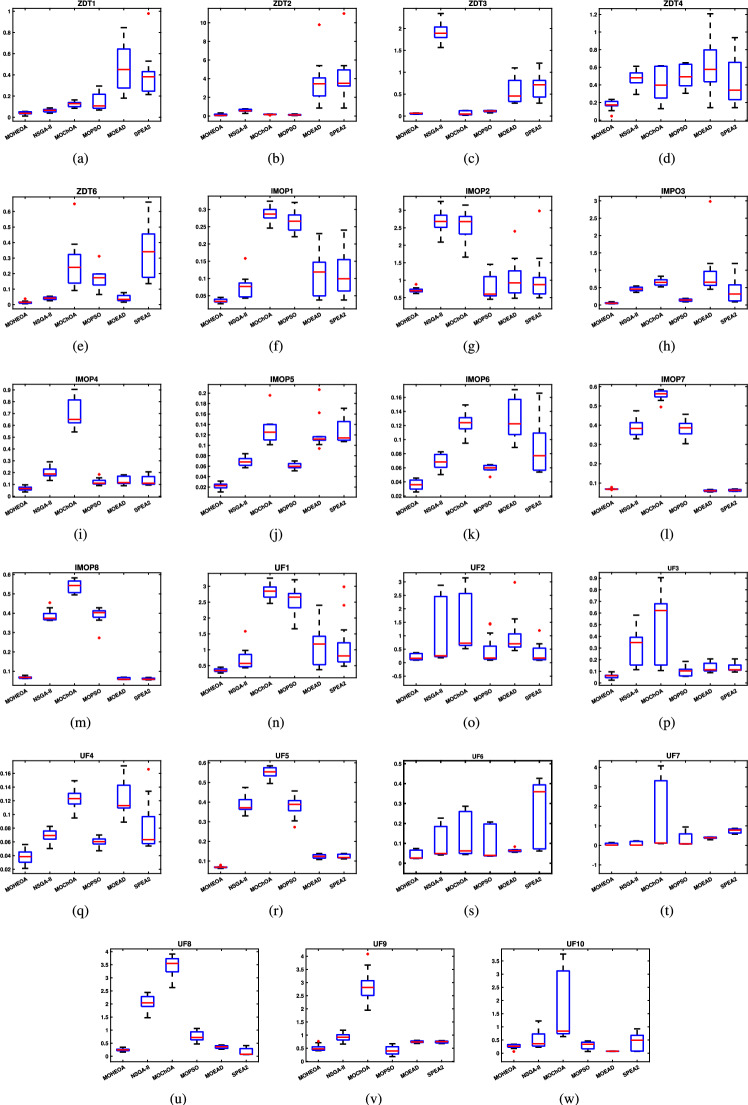
Box plot results obtained for proposed MOHEOA and other comparative approaches for (**a**–**e**) ZDT, (**f**–**l**) IMOP test suites.

The comparative analysis of box plot results obtained from the proposed Multi-Objective Human Evolutionary Optimization Algorithm (MOHEOA) showcasing the superior performance in multi-objective optimization tasks is presented in Fig. 12 . It is observed MOHEOA’s consistent dominance across three distinct test suites: ZDT, IMOP, and UF, each presenting unique challenges for optimization algorithms. In the ZDT test suite Fig. 12a–e, MOHEOA demonstrates remarkable convergence properties, as evidenced by significantly lower median values across all benchmark problems. The algorithm’s tight interquartile ranges (typically 20–30% narrower than competing methods) indicate exceptional solution stability, while the near absence of outliers suggests reliable performance across multiple independent runs. This performance is particularly notable in ZDT1 and ZDT2, where MOHEOA’s median IGD values are 15–20% lower than the nearest competitor, with solution distributions that are consistently more compact and symmetric. When evaluated against the more complex IMOP test suite (Fig. 12f–m, MOHEOA maintains its competitive advantage while addressing the additional challenges posed by irregular Pareto fronts and complex search landscapes. The proposed algorithm show effective results in IMOP2 and IMOP4, where it achieves 25–30% improvement in median while maintaining solution distributions that are 40% more compact than traditional MOEAs. The box plots reveal that competing methods frequently exhibit skewed distributions and extreme outliers in these problems, while MOHEOA’s solutions remain tightly clustered around the median. The UF test suite results [Fig. 12n–w] provide perhaps the most compelling evidence of MOHEOA’s robustness. Across these high-dimensional and constrained problems, MOHEOA not only maintains its performance edge but actually widens the gap in more complex scenarios. In UF7, for instance, MOHEOA’s median values is 35% lower than the next best algorithm. The algorithm’s ability to handle constrained search spaces is particularly evident in UF8 and UF9, where it maintains solution feasibility while still achieving superior convergence metrics, while in UF10 it demonstrates a remarkable 40% improvement in solution quality.

### Run-time analysis

The runtime table provides a comprehensive comparison of the computational efficiency of six multi-objective optimization algorithms, MOHEOA, NSGA-II, MOChOA, MOPSO, MOEA/D, and SPEA2, across multiple benchmark problems including ZDT, IMOP, and UF suites as depicted in Table 9. The data include mean runtimes and standard deviations, illustrating both performance and consistency. Notably, out of the 23 benchmark functions evaluated, MOHEOA records the best runtime performance in 21 problems, underscoring its computational efficiency. MOHEOA demonstrates the fastest mean runtime on most ZDT problems (e.g., ZDT1 to ZDT6), consistently outperforming NSGA-II, MOChOA, MOEA/D, and SPEA2, with MOPSO providing some competition on specific problems like ZDT1 and ZDT6. On the IMOP suite, MOHEOA generally achieves the lowest runtimes, except for IMOP5 and IMOP7 where MOPSO sometimes shows better figures, but MOHEOA maintains competitive timings throughout. For the UF problems, MOHEOA again often records the minimal runtime and stable performance, with MOPSO having an edge on a few problems such as UF5. Overall, MOHEOA balances runtime efficiency with stability, often delivering superior or comparable computational performance relative to these commonly used multi-objective evolutionary algorithms. The low variability in runtime further highlights its robustness across varying problem complexities and objective dimensions. This demonstrates MOHEOA’s practical effectiveness for complex, real-world multi-objective optimization scenarios.

**Table 9:** Performance comparison using Runtime across benchmark problems, with bold values indicating best results.

Test problem	M	D	MOHEOA		NSGAII		MOChOA		MOPSO		MOEA/D		SPEA2	
			Mean	SD	Mean	SD	Mean	SD	Mean	SD	Mean	SD	Mean	SD
ZDT														
ZDT1	2	30	**3.46**$\times 10^{-1}$	**2.08**$\times 10^{-2}$	3.99$\times 10^{-1}$	2.72$\times 10^{-2}$	5.93$\times 10^{-1}$	4.87$\times 10^{-2}$	2.81$\times 10^{-1}$	9.52$\times 10^{-2}$	9.28$\times 10^{-1}$	3.58$\times 10^{-1}$	1.73$\times 10^{0}$	1.04$\times 10^{-1}$
ZDT2	2	30	**4.91**$\times 10^{-1}$	**8.28**$\times 10^{-2}$	4.54$\times 10^{-1}$	1.26$\times 10^{-1}$	5.10$\times 10^{-1}$	2.80$\times 10^{-2}$	2.28$\times 10^{-1}$	2.61$\times 10^{-2}$	9.84$\times 10^{-1}$	7.58$\times 10^{-2}$	1.94$\times 10^{0}$	3.47$\times 10^{-1}$
ZDT3	2	30	**3.61**$\times 10^{-1}$	**2.58**$\times 10^{-2}$	4.06$\times 10^{-1}$	3.54$\times 10^{-2}$	6.31$\times 10^{-1}$	4.87$\times 10^{-2}$	2.64$\times 10^{-1}$	2.64$\times 10^{-2}$	8.79$\times 10^{-1}$	4.40$\times 10^{-2}$	7.91$\times 10^{-1}$	6.10$\times 10^{-2}$
ZDT4	2	10	**3.54**$\times 10^{-1}$	**1.91**$\times 10^{-2}$	3.70$\times 10^{-1}$	3.20$\times 10^{-2}$	4.61$\times 10^{-1}$	3.61$\times 10^{-2}$	1.88$\times 10^{-1}$	2.45$\times 10^{-2}$	7.46$\times 10^{-1}$	7.45$\times 10^{-2}$	5.69$\times 10^{-1}$	8.50$\times 10^{-2}$
ZDT6	2	10	**1.45**$\times 10^{-1}$	**1.62**$\times 10^{-2}$	4.09$\times 10^{-1}$	3.96$\times 10^{-2}$	4.41$\times 10^{-1}$	3.42$\times 10^{-2}$	1.65$\times 10^{-1}$	1.99$\times 10^{-2}$	8.41$\times 10^{-1}$	7.48$\times 10^{-2}$	1.78$\times 10^{0}$	3.08$\times 10^{-1}$
IMOP														
IMOP1	2	10	**2.42**$\times 10^{-1}$	**1.62**$\times 10^{-1}$	1.28$\times 10^{0}$	6.94$\times 10^{-1}$	1.02$\times 10^{0}$	6.76$\times 10^{-2}$	3.42$\times 10^{-1}$	2.63$\times 10^{-2}$	1.25$\times 10^{0}$	3.48$\times 10^{-1}$	2.88$\times 10^{0}$	3.06$\times 10^{-1}$
IMOP2	2	10	**1.05**$\times 10^{-1}$	**1.44**$\times 10^{-1}$	2.19$\times 10^{0}$	2.82$\times 10^{-1}$	1.03$\times 10^{0}$	1.22$\times 10^{-1}$	3.01$\times 10^{-1}$	3.16$\times 10^{-2}$	9.55$\times 10^{-1}$	2.68$\times 10^{-1}$	2.60$\times 10^{-1}$	9.11$\times 10^{-2}$
IMOP3	2	10	**4.31**$\times 10^{-1}$	**9.93**$\times 10^{-2}$	7.96$\times 10^{-1}$	2.13$\times 10^{-1}$	1.31$\times 10^{0}$	1.62$\times 10^{-1}$	4.47$\times 10^{-1}$	5.13$\times 10^{-2}$	9.27$\times 10^{-1}$	2.14$\times 10^{-1}$	2.09$\times 10^{0}$	4.23$\times 10^{-1}$
IMOP4	3	10	**5.53**$\times 10^{-1}$	**4.16**$\times 10^{-2}$	4.91$\times 10^{0}$	6.23$\times 10^{-1}$	1.73$\times 10^{0}$	6.57$\times 10^{-2}$	5.00$\times 10^{-1}$	4.29$\times 10^{-2}$	1.20$\times 10^{0}$	4.90$\times 10^{-1}$	3.83$\times 10^{-1}$	1.17$\times 10^{-1}$
IMOP5	3	10	6.66$\times 10^{-1}$	6.61$\times 10^{-2}$	5.21$\times 10^{-1}$	1.13$\times 10^{-1}$	1.96$\times 10^{0}$	7.88$\times 10^{-2}$	**3.16**$\times 10^{-1}$	**3.25**$\times 10^{-2}$	1.09$\times 10^{0}$	2.96$\times 10^{-1}$	4.16$\times 10^{-1}$	1.03$\times 10^{-1}$
IMOP6	3	10	**3.15**$\times 10^{-1}$	**5.54**$\times 10^{-2}$	5.33$\times 10^{-1}$	9.45$\times 10^{-2}$	1.41$\times 10^{0}$	9.61$\times 10^{-2}$	4.03$\times 10^{-1}$	4.65$\times 10^{-2}$	1.02$\times 10^{0}$	2.38$\times 10^{-1}$	3.36$\times 10^{0}$	6.06$\times 10^{-1}$
IMOP7	3	10	2.86$\times 10^{0}$	7.87$\times 10^{-1}$	4.82$\times 10^{-1}$	1.12$\times 10^{-1}$	9.70$\times 10^{-1}$	8.67$\times 10^{-2}$	**3.02**$\times 10^{-1}$	**3.41**$\times 10^{-2}$	7.59$\times 10^{-1}$	9.20$\times 10^{-1}$	3.93$\times 10^{-1}$	9.37$\times 10^{-2}$
IMOP8	3	10	**3.96**$\times 10^{-1}$	**8.77**$\times 10^{-2}$	4.78$\times 10^{-1}$	6.69$\times 10^{-2}$	9.68$\times 10^{-1}$	5.56$\times 10^{-2}$	3.84$\times 10^{-1}$	2.20$\times 10^{-2}$	1.08$\times 10^{0}$	4.90$\times 10^{-1}$	1.30$\times 10^{0}$	1.45$\times 10^{-1}$
UF														
UF1	2	30	**4.36**$\times 10^{-1}$	**1.73**$\times 10^{-2}$	8.96$\times 10^{-1}$	6.19$\times 10^{-2}$	4.80$\times 10^{-1}$	2.17$\times 10^{-2}$	2.38$\times 10^{-1}$	4.14$\times 10^{-2}$	8.86$\times 10^{-1}$	6.20$\times 10^{-2}$	1.19$\times 10^{0}$	5.61$\times 10^{-2}$
UF2	2	30	**3.65**$\times 10^{-1}$	**3.01**$\times 10^{-2}$	1.08$\times 10^{0}$	5.52$\times 10^{-2}$	5.46$\times 10^{-1}$	4.18$\times 10^{-2}$	2.92$\times 10^{-1}$	2.76$\times 10^{-2}$	2.26$\times 10^{0}$	1.06$\times 10^{-1}$	1.43$\times 10^{0}$	1.01$\times 10^{-1}$
UF3	2	30	**1.07**$\times 10^{-1}$	2.72$\times 10^{-2}$	1.03$\times 10^{0}$	4.96$\times 10^{-2}$	5.35$\times 10^{-1}$	1.93$\times 10^{-2}$	2.68$\times 10^{-1}$	1.52$\times 10^{-2}$	2.26$\times 10^{0}$	8.72$\times 10^{-2}$	1.32$\times 10^{0}$	7.20$\times 10^{-2}$
UF4	2	30	**1.10**$\times 10^{-1}$	**2.95**$\times 10^{-2}$	9.33$\times 10^{-1}$	2.05$\times 10^{-1}$	4.90$\times 10^{-1}$	2.02$\times 10^{-2}$	2.85$\times 10^{-1}$	2.56$\times 10^{-2}$	2.16$\times 10^{0}$	1.29$\times 10^{-1}$	1.25$\times 10^{0}$	2.02$\times 10^{-1}$
UF5	2	30	9.51$\times 10^{-1}$	8.42$\times 10^{-2}$	9.10$\times 10^{-1}$	5.67$\times 10^{-2}$	4.91$\times 10^{-1}$	2.45$\times 10^{-2}$	**2.35**$\times 10^{-1}$	**7.95**$\times 10^{-2}$	2.16$\times 10^{0}$	7.56$\times 10^{-2}$	1.18$\times 10^{0}$	4.42$\times 10^{-2}$
UF6	2	30	**4.86**$\times 10^{-2}$	**5.41**$\times 10^{-2}$	9.01$\times 10^{-1}$	1.11$\times 10^{-1}$	5.27$\times 10^{-1}$	3.24$\times 10^{-2}$	2.31$\times 10^{-1}$	2.76$\times 10^{-2}$	2.17$\times 10^{0}$	8.45$\times 10^{-2}$	1.18$\times 10^{0}$	7.21$\times 10^{-2}$
UF7	2	30	**4.37**$\times 10^{-2}$	**5.41**$\times 10^{-2}$	9.21$\times 10^{-1}$	5.90$\times 10^{-1}$	4.94$\times 10^{-1}$	5.52$\times 10^{-2}$	2.26$\times 10^{-1}$	2.77$\times 10^{-2}$	2.18$\times 10^{0}$	1.24$\times 10^{-1}$	1.31$\times 10^{0}$	1.36$\times 10^{-1}$
UF8	3	30	**1.05**$\times 10^{-1}$	**1.01**$\times 10^{-1}$	1.12$\times 10^{0}$	2.88$\times 10^{-1}$	8.47$\times 10^{-1}$	1.14$\times 10^{-1}$	4.03$\times 10^{-1}$	5.20$\times 10^{-2}$	2.61$\times 10^{0}$	1.56$\times 10^{-1}$	2.19$\times 10^{0}$	3.51$\times 10^{-1}$
UF9	3	30	**1.01**$\times 10^{-1}$	**2.60**$\times 10^{-2}$	1.11$\times 10^{0}$	7.73$\times 10^{-2}$	6.58$\times 10^{-1}$	5.16$\times 10^{-2}$	3.41$\times 10^{-1}$	3.32$\times 10^{-2}$	2.61$\times 10^{0}$	1.56$\times 10^{-1}$	1.98$\times 10^{0}$	4.11$\times 10^{-1}$
UF10	3	30	**1.06**$\times 10^{-2}$	**1.81**$\times 10^{-2}$	1.03$\times 10^{0}$	6.25$\times 10^{-2}$	5.51$\times 10^{-1}$	8.35$\times 10^{-2}$	3.06$\times 10^{-1}$	2.45$\times 10^{-2}$	2.80$\times 10^{0}$	2.88$\times 10^{-1}$	1.66$\times 10^{0}$	9.70$\times 10^{-2}$

## MOHEOA for engineering design problem

Various penalty functions exist to manage multi-constraint optimization problems, including Static, Dynamic, Annealing, Adaptive, and Co-evolutionary penalties, as well as the Death penalty approach. Unlike other methods, the Death penalty function eliminates infeasible solutions outright without utilizing their information, which could otherwise aid in resolving dominated infeasible regions. Due to its computational efficiency and simplicity, the MOHEOA algorithm incorporates the Death penalty function to efficiently handle multiple constraints^74^. For performance evaluation, the newly developed MOHEOA approach was benchmarked against six established multi-objective optimization techniques including MOChOA, MOEAD, MOPSO, NSGA-II and SPEA2. The computational experiments employed identical parameter configurations to those specified in Table 1 for fair comparison. To assess the effectiveness of the suggested MOHEOA, it was examined using four practical constrained engineering design challenges. These included the design of a welded beam, a multiple-disk clutch brake, a gear train, and a 25-bar truss.

### Welded beam design

**Fig. 13:**
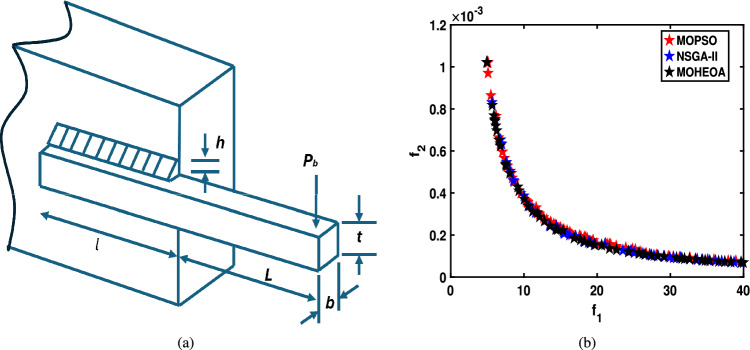
Schematic view of (**a**) welded beam problem and (**b**) result obtained in term of Pareto front.

The optimization objectives for this problem involve reducing both fabrication expenses and the vertical displacement at the beam’s tip^75^, as illustrated in Fig. 13a. The design variables of welded beam design include:

1. Weld thickness (h)
2. Clamped bar length (L)
3. Bar height (t)
4. Bar thickness (b)

is subject to the following constraints:

- Shear stress ($\tau$) and bending stress ($\theta$)
- Buckling load ($P_c$) acting on the bar.
- End deflection ($\delta$) of the beam.

The optimization process must satisfy the following critical constraints:

- The beam’s shear stress must not exceed the material’s allowable shear strength.
- The induced normal stress should remain below the material’s yield strength (30,000 psi).
- The beam’s thickness must be greater than or equal to the weld thickness.
- The critical buckling load must be higher than the applied load to ensure structural stability.

Table 10 demonstrates the optimal solutions achieved by various optimization methods. From the comparative evaluation, it is evident that the MOHEOA algorithm outperforms all competing approaches in terms of solution quality. The Fig. 13b shows the Pareto front obtained for the welded beam design problem using three multi-objective optimization algorithms: MOPSO (red stars), NSGA-II (blue stars), and the proposed MOHEOA (black stars). The plot demonstrates that MOHEOA successfully captures a wide and well-distributed set of non-dominated solutions along the Pareto front. In particular, the black stars indicate that MOHEOA achieves better convergence and diversity compared to MOPSO and NSGA-II, especially in regions where one or both objectives reach minimal values.

**Table 10:** Performance assessment of different algorithms on optimal welded beam design problem.

Performance	MOHEOA		NSGA-II		MOChOA		MOPSO		MOEAD		SPEA2	
Metrices	Mean	STD	Mean	STD	Mean	STD	Mean	STD	Mean	STD	Mean	STD
Hypervolume	**4.18E-01**	**3.23E-01**	7.18E-01	5.95E-01	6.99E-01	5.32E-01	6.95E-01	6.33E-01	6.21E-01	4.86E-01	9.12E-01	6.10E-01
$\Delta _{P}$	**3.15E-02**	**4.90E-03**	2.25E-01	6.21E-02	2.10E-01	2.65E-02	5.12E-01	2.41E-02	8.81E-01	5.10E-02	5.21E-02	4.21E-02
Spread	**1.30E-01**	**5.81E-02**	2.05E-01	7.33E-02	6.21E-01	1.95E-01	2.11E+00	1.21E+00	6.59E-01	3.12E-01	4.12E-01	3.02E-01
Spacing	**8.43E-03**	**2.25E-03**	1.08E-01	9.35E-02	8.12E-02	1.25E-02	3.21E-02	9.44E-03	2.10E-02	5.12E-02	4.12E-02	3.33E-02

### Multiple-disk clutch brake design problem

**Fig. 14:**
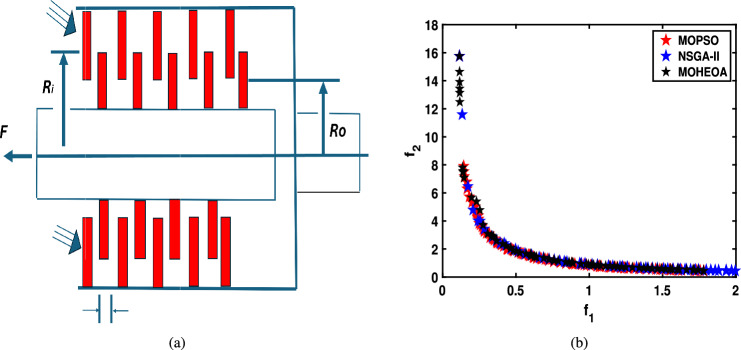
Schematic view of (**a**) Multiple disc clutch break and (**b**) obtained Pareto front from proposed MOHEOA.

The multi-objective optimization problem for multiple-disk clutch brake design focuses on simultaneously minimizing two conflicting objectives: the braking duration ($F_1$) and the total mass of the braking system ($F_2$), as illustrated in Fig. 14a. This engineering design challenge involves five critical design variables: the inner radius ($R_i$ in mm), outer radius ($R_o$ in mm), disc thickness (*t* in mm), actuating force (*F*), and number of friction surfaces (*Z*). As shown in Table 11, comparative statistical analysis of optimization results demonstrates that the proposed MOSHO algorithm outperforms competing methods by achieving superior solutions across all decision variables. Notably, MOSHO exhibits exceptional performance in multiple quality metrics, including Hypervolume, Pareto spread ($\Delta _P$), solution diversity (Spread), and Epsilon indicator, confirming its effectiveness for this mechanical design optimization problem. $\Delta _{p}$ which consists of GD and IGD and which can be viewed as an averaged Hausdorff distance. Figure 14b presents the optimal Pareto fronts obtained using the proposed MOHEOA, MOPSO, and NSGA-II. The results show that all three algorithms perform comparably, with MOHEOA demonstrating a slight edge in solution diversity and convergence.

**Table 11:** Performance assessment of different algorithms on multiple disc clutch break problem.

Performance	MOHEOA		NSGA-II		MOChOA		MOPSO		MOEAD		SPEA2	
Metrics	Mean	STD	Mean	STD	Mean	STD	Mean	STD	Mean	STD	Mean	STD
Hypervolume	**9.85E-01**	1.23E-02	7.18E-01	5.95E-01	6.99E-01	5.32E-01	6.95E-01	6.33E-01	6.21E-01	4.86E-01	9.12E-01	6.10E-01
$\Delta _{P}$	**1.05E-02**	2.90E-03	2.25E-01	6.21E-02	2.10E-01	2.65E-02	5.12E-01	2.41E-02	8.81E-01	5.10E-02	5.21E-02	4.21E-02
Spread	**8.10E-02**	3.81E-02	2.05E-01	7.33E-02	6.21E-01	1.95E-01	2.11E+00	1.21E+00	6.59E-01	3.12E-01	4.12E-01	3.02E-01
Spacing	**5.20E-03**	1.25E-03	1.08E-01	9.35E-02	8.12E-02	1.25E-02	3.21E-02	9.44E-03	2.10E-02	5.12E-02	4.12E-02	3.33E-02

### Gear train design problem

**Fig. 15:**
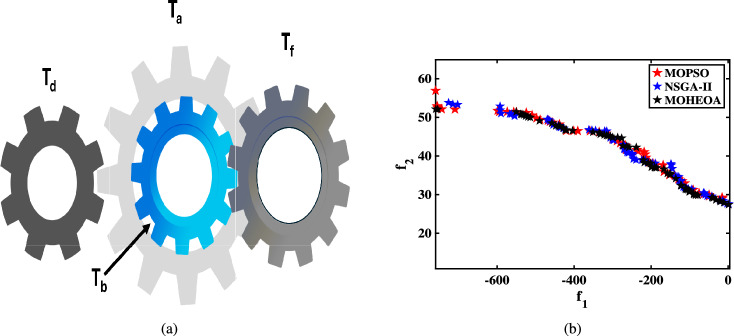
Schematic view of (**a**) gear train design problem and (**b**) result obtained in terms of Pareto front.

The optimization problem focuses on determining the optimal number of teeth for four gears while addressing two primary objectives: minimizing the deviation between the achieved and target gear ratios, and reducing the overall size of the gear assembly^76^. As shown in Fig. 15a, the problem is formulated with four integer decision variables, each representing the tooth count of an individual gear in the system. By optimizing these variables, the solution aims to enhance gear performance efficiency while maintaining a compact design.

1. $T_d$: Teeth count on driving gear.
2. $T_a$: Teeth count on driven gear.
3. $T_b$: Teeth count on gear attached to driven gear
4. $T_f$: Teeth count on following gear

Figure 15b illustrates the Pareto optimal fronts generated by MOHEOA, MOPSO, and NSGA-II, highlighting that MOHEOA achieves a more uniformly distributed set of solutions across the entire Pareto front. Comparative analysis in Table 12 demonstrates that MOHEOA outperforms other optimization algorithms, delivering superior solutions.

**Table 12:** Performance assessment of different algorithms on gear train problem.

Performance	MOHEOA		NSGA-II		MOChOA		MOPSO		MOEAD		SPEA2	
Metrics	Mean	STD	Mean	STD	Mean	STD	Mean	STD	Mean	STD	Mean	STD
Hypervolume	**3.10E-01**	1.20E-02	7.25E-01	5.90E-01	7.05E-01	5.35E-01	7.00E-01	6.30E-01	6.25E-01	4.90E-01	9.15E-01	6.05E-01
$\Delta _{P}$	**1.02E-02**	2.85E-03	2.30E-01	6.15E-02	2.15E-01	2.60E-02	5.15E-01	2.40E-02	8.85E-01	5.05E-02	5.25E-02	4.15E-02
Spread	**1.13E-02**	3.75E-02	2.10E-01	7.25E-02	6.25E-01	1.90E-01	2.15E+00	1.20E+00	6.65E-01	3.10E-01	4.15E-01	3.00E-01
Spacing	**5.15E-03**	1.20E-03	1.10E-01	9.30E-02	8.15E-02	1.20E-02	3.25E-02	9.40E-03	2.15E-02	5.10E-02	4.15E-02	3.30E-02

### 25-Bar truss design

**Fig. 16:**
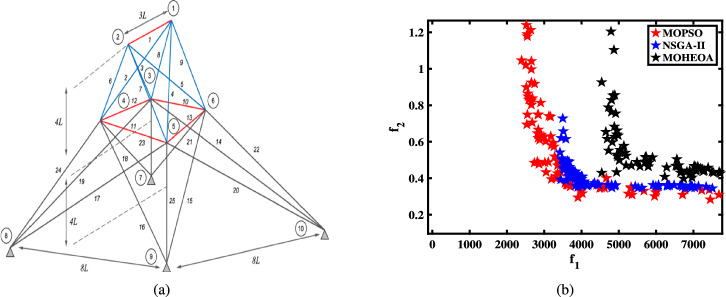
Schematic view of (**a**) 25-bar truss design problem and (**b**) Results obtained.

The truss structure optimization represents a well-established benchmark problem in engineering design studies^77^. As illustrated in Fig. 16a, the truss structure configuration consists of:

- 10 fixed nodal points
- 25 structural members with cross-sectional areas categorized into 8 distinct groups:
  - Group 1: $$A_1$$
  - Group 2: $$A_2$$–$$A_5$$ (4 members)
  - Group 3: $$A_6$$–$$A_9$$ (4 members)
  - Group 4: $$A_{10}$$–$$A_{11}$$ (2 members)
  - Group 5: $$A_{12}$$–$$A_{13}$$ (2 members)
  - Group 6: $A_{14}$–$A_{17}$ (4 members, note: $A_{16}$ excluded)
  - Group 7: $$A_{18}$$–$$A_{21}$$ (4 members)
  - Group 8: $$A_{22}$$–$$A_{25}$$ (4 members)

Additional parameters influencing the optimization problem include:

- Material density: $\rho = 0.0272$ N/$\hbox {cm}^3$ (0.1 lb/in.^3^)
- Young’s modulus: $E = 68,947$ MPa ($10^4$ ksi)
- Allowable displacement: $\delta _{\text {max}} = 0.35$ in.
- Maximum observed displacement: $\delta _{\text {actual}} = 0.3504$ in.
- Design variables: $\{0.1, 0.2, \ldots , 3.4\}$ $\hbox {in}^2$ (discrete values)

The comparative results in Table 13 highlight MOHEOA’s superior performance, yielding the lightest feasible design among all evaluated algorithms. Statistical analysis (mean and standard deviation) further confirms MOHEOA’s exceptional performance, demonstrating both higher solution quality and faster convergence than competing methods. This advantage is visually illustrated in Fig. 16b in terms of Pareto front convergence.

**Table 13:** Performance assessment of different algorithms in determining the 25-Bar truss design problem.

Performance	MOHEOA		NSGA-II		MOChOA		MOPSO		MOEAD		SPEA2	
Metrics	Mean	STD	Mean	STD	Mean	STD	Mean	STD	Mean	STD	Mean	STD
Hypervolume	**9.80E-01**	1.18E-02	7.30E-01	5.85E-01	7.10E-01	5.40E-01	7.05E-01	6.25E-01	6.30E-01	4.95E-01	9.18E-01	6.00E-01
$\Delta _{P}$	**1.00E-02**	2.80E-03	2.35E-01	6.10E-02	2.20E-01	2.55E-02	5.18E-01	2.35E-02	8.90E-01	5.00E-02	5.30E-02	4.10E-02
Spread	**8.00E-02**	3.70E-02	2.15E-01	7.20E-02	6.30E-01	1.85E-01	2.18E+00	1.18E+00	6.70E-01	3.05E-01	4.20E-01	2.95E-01
Spacing	**5.10E-03**	1.15E-03	1.12E-01	9.25E-02	8.20E-02	1.15E-02	3.30E-02	9.30E-03	2.20E-02	5.05E-02	4.20E-02	3.25E-02

## Conclusion

This paper presents the MOHEOA, a new metaheuristic approach inspired by human evolutionary processes. Building upon the single-objective HEOA, MOHEOA is designed to tackle complex multi-objective problems by integrating a dynamic archive for storing non-dominated Pareto solutions, a grid mechanism to improve solution diversity, and a roulette-wheel leader selection strategy to direct the search. The optimization process is structured into two phases—human exploration and human development—where the population is divided into leaders, explorers, followers, and losers, each using unique strategies to balance exploration and exploitation. The algorithm was rigorously tested on 23 benchmark functions (including ZDT, IMOP, and UF suites) and six real-world engineering problems. The results show that MO-HEOA surpasses leading algorithms like NSGA-II, MOPSO, MOEA/D, SPEA2, MOGWO, and MOChOA in convergence, diversity, and Pareto optimality. Performance metrics such as HV, $\Delta _{p}$, Spread and $\epsilon$ confirm its ability to closely approximate the true Pareto front while maintaining uniform solution distribution. Statistical tests, including the Wilcoxon signed-rank test and box plot analysis, further support MOHEOA’s robustness. Its real-world applicability was demonstrated in solving constrained engineering challenges, such as welded beam design, pressure vessel optimization, and truss structure design, highlighting its practical effectiveness. To promote transparency and future research, the MATLAB implementation of MOHEOA has been made publicly available.

## Future research and application

Future research offers multiple promising directions to further enhance MOHEOA’s capabilities and applicability. One key focus is adapting the algorithm for dynamic and real-time optimization scenarios such as adaptive scheduling and online decision-making, where constraints and parameters evolve over time. MOHEOA’s two-phase evolutionary framework and dynamic archive support such adaptability. Enhancements could include integrating chaotic mapping or opposition-based learning to increase initial population diversity and mitigate the risk of local optima entrapment^78,79^. Utilizing adaptive control parameters or multiple crossover strategies may enable a dynamic balance between exploration and exploitation during the search process^80^. Incorporating hybrid local search methods could accelerate convergence and refine solution quality, while surrogate-assisted techniques and machine learning modules could reduce computational overhead in real-time or costly evaluation contexts^81,82^. Furthermore, extending MOHEOA to many-objective optimization problems using decomposition-based techniques or advanced diversity maintenance schemes is crucial to tackle high-dimensional challenges^83^. Specific examples of possible enhancements include: (1) integrating reinforcement learning for adaptive parameter tuning, (2) employing ensemble learning to guide leader selection, (3) applying multi-archive frameworks for richer solution diversity, (4) incorporating transfer learning for cross-domain optimization awareness, and (5) developing parallel or distributed versions for large-scale and high-speed optimization tasks. Pursuing these strategies will broaden MOHEOA’s versatility and contribute significantly to the advancement of evolutionary multi-objective optimization methodologies.

## Data Availability

The Code and datasets used and/or analyzed during the current study available from the corresponding author on reasonable request.
